# The impact of osteogenesis imperfecta severity on oral health-related quality of life in Spain: a cross-sectional study

**DOI:** 10.1186/s13023-024-03096-y

**Published:** 2024-03-08

**Authors:** Amira Ahmed Elfituri, Manuel Joaquín De Nova, Mohammadamin Najirad

**Affiliations:** 1 Faculty of Dentistry, Complutense University, Pza. Ramon y Cajal, Moncloa-Aravaca, Madrid Spain; 2 Department of Orthodontics, Faculty of Dentistry, University of Toronto, Toronto, Canada

**Keywords:** Osteogenesis Imperfecta, Oral health-related quality of life, Oral health impact profile

## Abstract

**Background:**

Osteogenesis imperfecta (OI) affects dental and craniofacial development; therefore, it can influence oral health-related quality of life (OHRQoL). The objective of this study was to explore the influence of the severity of OI on OHRQoL in adults older than eighteen years residing in Spain.

**Method:**

Adults with OI were recruited from the Spanish Association of Crystal Bone (AHUCE) foundation. OHRQoL was evaluated using the Spanish version of the Oral Health Impact Profile questionnaire (OHIP-14sp), oral hygiene habits, and a dental care survey. Clinical and radiological dental examinations were performed to evaluate the patients’ oral conditions.

**Results:**

A total of 65 adults (*n* = 46 females) aged between nineteen and sixty-two years who were diagnosed with OI and classified as type I, III, and IV (*n* = 20, 14, and 31, respectively) participated in this research. The total OHIP-14sp scores were significantly greater (worse) for type III (23 [SD = 10]) and type IV (21.4 [SD = 12]) than for type I (13.8 [SD = 6]) (*P* < 0.05). The negative impact of OHRQoL was due to the association of type III OI with all domains except for the handicap domain, while type IV OI was associated with the physical disability, social disability, and handicap domains (*P* < 0.05 for all).

**Conclusion:**

The severity of OI negatively impacted OHRQoL in adults. This association was statistically significant.

**Supplementary Information:**

The online version contains supplementary material available at 10.1186/s13023-024-03096-y.

## Introduction

Osteogenesis imperfecta (OI) is known as brittle bone disease and is a rare, chronic, and currently noncurable disease characterized by inadequate formation of bone tissue due to a lack of collagen (mainly Col1A1 and Col1A2) or poor quality [1]. The incidence of OI is estimated to be between 10 in 100,000 newborns, and the approximate prevalence is 5 in 100,000 people [2]. This estimate is within the lower limit, as mild forms of the disease are often not diagnosed. In Spain, there could be a minimum of 2700 people affected by any type of OI [3]. There is a growing trend among researchers interested in this field to refer to it as “osteogenesis imperfecta syndrome”, which is characterized by low bone mass, bone fragility, joint laxity, hearing loss, blue sclera, and a wide spectrum of clinical severity, from almost straight bones and very few fractures to others with multiple fractures, including intrauterine fracture [4]. Since 2006, OI has been known to be caused by several mutations in collagen-related genotypes, resulting in over 27 types, each with a unique inheritance pattern. Despite being widely used, the new genomic classification system is still under debate. The large number of types complicates the classification of the disease; hence, for simplicity of evaluation, we still follow the old classification described by Sillence [5]. OI type I is the mildest and most common OI type with the absence of major bone deformities. OI type II usually results in prenatal or perinatal death. type III is the most severe type with a high degree of deformity and a very short stature, and OI type IV has characteristics that are intermediate in disease severity between OI types I and III [5]. The goal of OI treatment is to alleviate symptoms and support normal function. Moreover, by decreasing bone turnover and improving bone mineral density, bisphosphonates are regarded as the most effective treatment for minimizing fractures and pain [2]. Bisphosphonates are regarded as the most effective treatment [6]. The most frequent complication in patients receiving bisphosphonate treatment is osteonecrosis of the jaw (ONJ) [7], which can occur following any surgical dental treatment. The risk for the development of ONJ is greater in individuals receiving intravenous bisphosphonate than in individuals receiving oral bisphosphonate therapy [8].

Individuals with OI may have malocclusion or misalignment of the teeth and jaw, especially in people with more severe forms of OI [9]. Dental problems such as dentinogenesis imperfecta (DI), agenesis, impacted teeth, and ectopic eruption contribute to malocclusion in this population [10, 11]. These anomalies that develop in early life and worsen with age in the OI population can lead to difficulties chewing, biting, and speaking and can cause pain and discomfort [12].

Oral health has been recognized as part of overall health and an important component of quality of life [13]. Perhaps, this has resulted in the development of the concept of “oral health-related quality of life” (OHRQoL), which includes five categories: functional factors, psychological factors, social factors, environmental factors, and the existence of discomfort or pain [14]. Najirad et al. (2018) demonstrated that the severity of OI impacts OHRQoL in adolescents aged 11 to 14 years. Scores were greater (i.e., worse) for type lll and IV than for type l. Differences were associated with a significantly greater grade of functional limitations in OI types lll and IV than in OI type I. In the abovementioned study, scores were similar between OI types in children aged 8 to 10 years [12]. Furthermore, according to Rizkallah and colleagues’ (2013) analysis of the peer assessment rating and discrepancy index, individuals with OI have significantly lower estimates for five malocclusion traits than does the general population, including anterior open bite, posterior open bite, anterior crossbite, posterior crossbite, and Angle classification III [15]. Understanding the relationship between the severity of OI and its impact on OHRQoL will help dentists and health care professionals provide more effective care and treatment strategies. They can tailor their approaches to address the specific needs and challenges faced by individuals with severe OI types, which may include more frequent dental check-ups, specialized oral care plans, or preventive measures to minimize dental complications [16]. This is the first study to carry out oral clinical examinations among adults above eighteen years old with OI in Spain. There are neither registrations nor published articles describing the oral conditions of Spanish adults with OI. Therefore, our objective was to study the oral status of adults with OI and to explore the impact of disease severity on OHRQoL. We hypothesized that severe OI types would have a greater negative impact on OHRQoL than would those less severe OI types.

## Materials and methods

Adults above eighteen years old with OI were recruited from the Spanish Association of Crystal Bones (AHUCE) Foundation [3], which serves individuals with a clinical and/or molecular diagnosis of OI in Spain. This was a cross-sectional study consisting of a questionnaire and clinical examination. Participants were divided according to their phenotype and clinical symptoms by the scientific committee of the abovementioned foundation into three groups (types I, III, and VI). In this study, type II OI patients were excluded due to the severity of the condition and high mortality rate. All participants who did not have a genetic test confirming the diagnosis of the disease were excluded before statistical analysis [2]. All participants were informed about the purpose of this study. Participation was voluntary, and each patient received a complete dental report free of charge. This investigation was approved by the San Carlos Clinical Hospital Committee for Medical and Health Research Ethics of Madrid, and all study participants provided informed consent.

### Questionnaire

An online self-completed questionnaire in concordance with the checklist for reporting results of internet E-surveys [17] was launched on the AHUCE website from April 2020 to April 2022. Only individuals with OI had access to the questionnaire. Several reminders were sent to the participants to complete the questionnaire. The questionnaire included demographic and medical history questions. As participants could complete the questionnaire more than once, a regular check for duplicate entries and data-cleaning procedures were employed to identify and remove any duplicate responses.

### Oral health impact profile questionnaire

The original questionnaire (OHIP-49) consists of 49 items that were developed based on a theoretical model by the World Health Organization (WHO) [18]; this questionnaire was subsequently adapted by Locker & Jokovic, (1996) and proved by Allen et al. (1999) [19, 20]. The original version was simplified to a 14-item scale (OHIP-14) [21], which is reliable despite being a short questionnaire. A Spanish version of the short form of the questionnaire was validated to assess the association between quality of life and oral health in elderly Chilean patients [22], and it has proven to be an accurate, valid, and reliable instrument for assessing oral health-related quality of life among adults in Spain [23]. For that reason, it is widely used in both cross-sectional and longitudinal studies and is internationally accredited (24–25). The questionnaire focuses on seven dimensions of impact (functional limitation, pain, psychological discomfort, physical disability, psychological disability, social disability, and handicap) for the determination of OHRQoL. The answers are coded according to the frequency of impact on OHRQoL into a 5-point Likert scale as never (score of 0), hardly ever (score of 1), occasionally (score of 2), fairly often (score of 3) and very often (score of 4). To obtain the score, values are added, with a minimum of 0 points and a maximum of 56 points for every patient. Therefore, low scores indicate better self-perceived QoL, and high scores indicate worse self-perceived QoL [21]. Additionally, this self-perception can be expressed as negative or positive. The questionnaire is available in English and Spanish (see Additional File 1).

#### Oral hygiene habits and dental care survey

The first author conducted a dental survey that included ten questions relating to personal oral hygiene behaviors, dental prophylaxis (maintenance), and the habit of visiting their dentist, i.e., dental checkups or in cases of complaints (see Additional File 2).

### Clinical and radiological dental examination

**Fig. 1:**
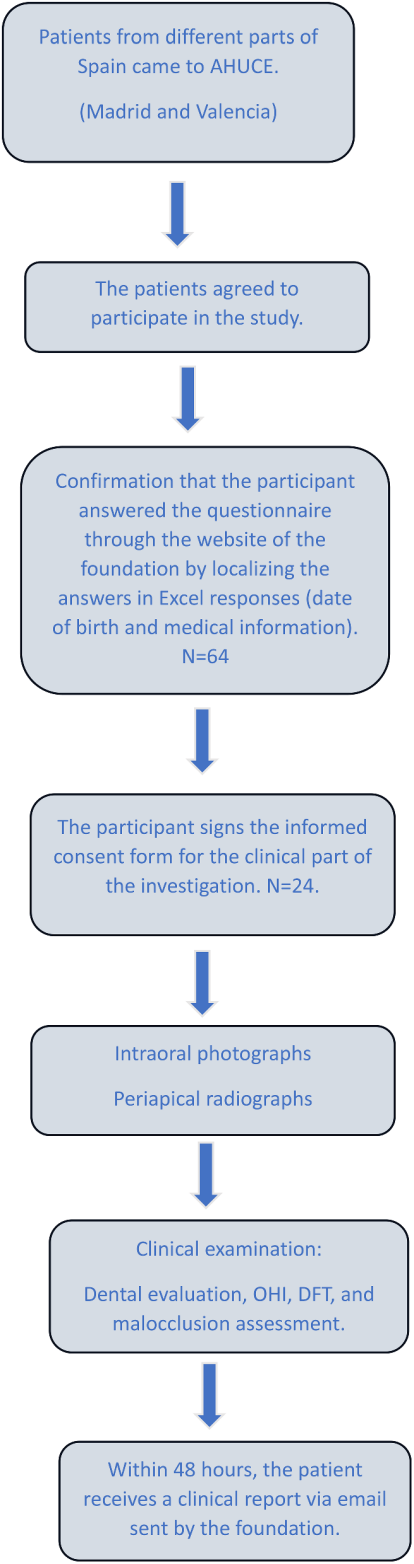
A flow chart illustrating the recruitment process

Oral examination and dental evaluation for malocclusions were performed by an orthodontist at AHUCE (Madrid and Valencia). The orthodontist was trained by an expert with more than 30 years of clinical experience with OI at University Complutense of Madrid Department of Dental Specialties. To assess intra-examiner reliability, 20 OI children with an average age of eleven not part of this study were randomly selected and re-examined at a 2-week after their first examination. The kappa used to measure inter-rater reliability for qualitative (categorical) items value was 0.915. Patients from different parts of Spain came to the foundation for psychological and rehabilitation treatment or for learning more about their disease. Phone call conversations were carried out with patients in advance of their visits to the foundation to give them the opportunity to participate in our study. People who agreed to participate in our study were asked to complete the questionnaire before the clinical examination (see Fig. 1: the study flow chart). As a part of the investigation, intraoral photos and a full-mouth periapical survey (using a portable dental X-ray machine and sensor) were taken instead of panoramic radiographs. Since this study was carried out during the COVID-19 pandemic, patients could not be scheduled for panoramic radiographs. Participation in the clinical examination was very low (37%) (n = 24). One of the participants was edentulous and was therefore not radiographed. The oral hygiene of the participants was evaluated based on the simplified oral hygiene index (OHI-S), which consists of two elements: a simplified debris index (DI-S) and a simplified calculus index (CI-S), each rated on a scale of 0 to 3 [26]. Adjusted, decayed and filled teeth (ADFT) were used for caries experience instead of decayed, missing, and filled teeth (DMFT) as the authors believe that the adjusted index addresses the causes of missing teeth. The ADFT index was previously employed in investigations of Down syndrome and OI [27, 28]. The scores were adjusted by dividing the total number of caries and fillings by the total number of teeth present at the time of dental examination to increase the accuracy of the caries experience. The ADFT index is a continuous variable ranging from 0 to 1 [28]. The presence of dentinogenesis imperfecta was determined by increased translucency of the enamel and blue-gray to brown discoloration of the teeth, in addition to the presence of abnormal radiographic signs such as pulp obliteration, short roots, thin roots, and cervical constriction [11]. Radiographs were used to determine the number of existing teeth, endodontically treated teeth, and implants. For statistical analysis, missing teeth and endodontically treated teeth were divided into three groups: 0–3, 4–7, and > 7. The registration did not include third molars. The use of removable dentures was also recorded. The assessment of malocclusion was performed in three planes: the sagittal, transverse, and vertical planes. In the sagittal plane, by using Angle classification, molars were recorded as 1 (class I), 2 (class III) or 3 (missing molars), or the presence of removable dentures recorded as “other”. Only one patient had molar class II, and this patient was classified into the “other” group in our main analysis. Crossbites (anterior and posterior) were registered with the mandibular molars in centric occlusion. Anterior crossbite in the case of maxillary incisors was palatally positioned to the mandibular incisors. In the transverse plane, posterior crossbite was recorded when maxillary molars were occluded in a lingual relationship. Single-tooth crossbite was not considered a type of crossbite. In the vertical plane, an open bite (anterior and posterior) was registered when there was a gap between the upper and lower teeth while the mandible was in centric occlusion.

### Data analyses

The statistical analysis of the data was performed with IBM SPSS Statistics version 29.0 for Windows. A summary of the characteristic data of the sample is shown in Table 1, contingency tables for the relationships between qualitative variables (CROSSTABS procedure). A chi-square test was used to assess the independence of two qualitative variables with small cell counts. The nonparametric Kruskal‒Wallis test (NPTESTS procedure) was used, with the OHIP-14 score and subscale score used as dependent variables for comparisons of quantitative variables between more than two groups. The Mann‒Whitney U test (nonparametric) was performed to assess the significance of differences between two groups. OHIP-14 scores and their constituent subscale scores were transformed into ordinal variables using their 33rd and 66th percentiles. Sex and age were identified as the minimum set of potential confounders to be included in the multivariate analyses. Multivariate ordinal logistic regression analyses were employed to estimate the total effect of OI type on the OHIP-14 score and its constituent domains.

**Table 1:** Characteristics of adults with OI and their oral condition

Type of OI	Mild OI type I	Moderate OI type IV	Severe OI type III	All	
*Sociodemographic characteristics*					
Enrollment number– n (%)	20 [31]	31 [47]	14 [21]	65 (100)	
Female	18 (90)	19 (61)	9 (64)	46 (70)	
Age– Average Range	29	35	35	-	
*Educational level– n (%)*					
Some college - high school or less	2 [10] − 6 [30]	8 [26] − 13 [41]	4 [29] − 7 [13]	14 [22] − 25 [39]	
College graduate– postgraduate	8 [40] − 4 [20]	7 [23] − 3 [10]	20 [14] − 1 [7]	17 [27] − 8 [12]	
Pertinent Medical Conditions					
*Genetic mutation– n (%)*					
Col1A1 - Col1A2	16 (80) − 2 [10]	19 (61) − 7 [23]	5 [36] − 4 [29]	40 (62) − 12 [19]	
Others– unsure	0 (0) − 2 [10]	1 [3] − 4 [13]	3 [21] − 2 [14]	4 [6] − 8 [13]	
*OI medications– n (%)*					
Bisphosphonate + other types of drugs– Yes	10 [13]	18 (58)	9 (64)	37 (57)	
Bisphosphonate	8 (80)	12 (67)	4 [45]	24 (65)	
Oral– IV	3 (37.5)– 5 (62.5)	1 (8.3)-11 (91.7)	1 [25]- 3 (75)	5 (20.8)– 19 (79.2)	
Denosumab (Prolia)	0 (0)	3 [17]	1 [11]	4 [11]	
Teriparatide (Forsteo)	1 [10]	0 (0)	1 [11]	2 [5]	
Other types of drugs	1 [10]	3 [17]	3 [33]	7 [19]	
Vit D intake– Yes	12 (60)	26 (84)	12 (86)	50 (77)	
Calcium intake– Yes	12 (60)	14 [45]	10 (72)	36 (55)	
*Oral Conditions*					
Enrollment number– n (%)	9 [36]	10 [44]	5 [20]	24 (100)	
DI (Yes)– n (%)	2 [22]	5 [13]	1 [20]	8 [33]	
Molar classification– n (%)					
Cl	6 (67)	3 [30]	0 (0)	9 [38]	
Clll	2 [22]	4 [40]	3 (60)	9 [38]	
Cll and neglected*	1 [11]	3 [30]	2 [40]	6 [24]	
Anterior open bite– n (%) Yes	0 (0)	2 [20]	3 (60)	5 [21]	
Posterior open bite– n (%) Yes	1 [11]	0 (0)	3 (60)	4 [17]	
Anterior crossbite– n (%) Yes	0 (0)	3 [30]	3 (60)	6 [25]	
Posterior crossbite– n (%) Yes	1 [11]	3 [30]	3 (60)	7 [29]	

Statistical tests between categorical variables and OI types (mild, moderate, and severe): chi-square test for contingency tables; Kruskal‒Wallis test for age; and oral survey with OI type as the independent variable. *Neglected: missing molars or removable dentures

## Results

Eighty individuals participated in this study, and fifteen were excluded because they had not undergone genetic testing. The remaining 65 individuals, of whom 46 were females, with a mean age of 42.6 years (range = 18–68 years), were classified according to their phenotype and clinical symptoms into three groups of OI types I, III, and IV (*n* = 20, 14, and 31, respectively). Thirty-nine of the participants had an education level of high school or less. There were no significant differences between the different types of OI concerning sociodemographic factors. The majority of the participants (*n* = 40) had the genetic mutation Col1A1, twelve had Col1A2, eight did not remember the type of genetic mutation, and four had other types of genetic mutations. The number of participants with the remaining genetic mutations was too small for statistical analysis, and this sample was analyzed in the “other” group (Table 1). More than half of the study population (*n* = 37) was treated with OI medications (bisphosphonate, denosumab, forsteo, and others) (*n* = 24, *n* = 4, *n* = 2, and *n* = 7, respectively). In our investigation, thirty of the participants had diseases other than OI. Three of them had diabetes mellitus (two patients with type III OI and one with type IV OI). The other three participants, with type IV OI, had asthma. One patient had a history of breast cancer with type IV OI. The patient underwent chemotherapy and radiotherapy 5 years prior. The oral condition of this patient was very poor (only one tooth in the mandible and seven teeth in the maxilla were present, all of which had severe horizontal bone loss), which may have contributed to worsening OHRQoL. The remaining participants had different diseases, such as hypertension, osteosclerosis, hypothyroidism, different heart problems, and blood diseases. We were unable to exclude these patients due to the rarity of the diseases and the small sample size.

**Fig. 2:**
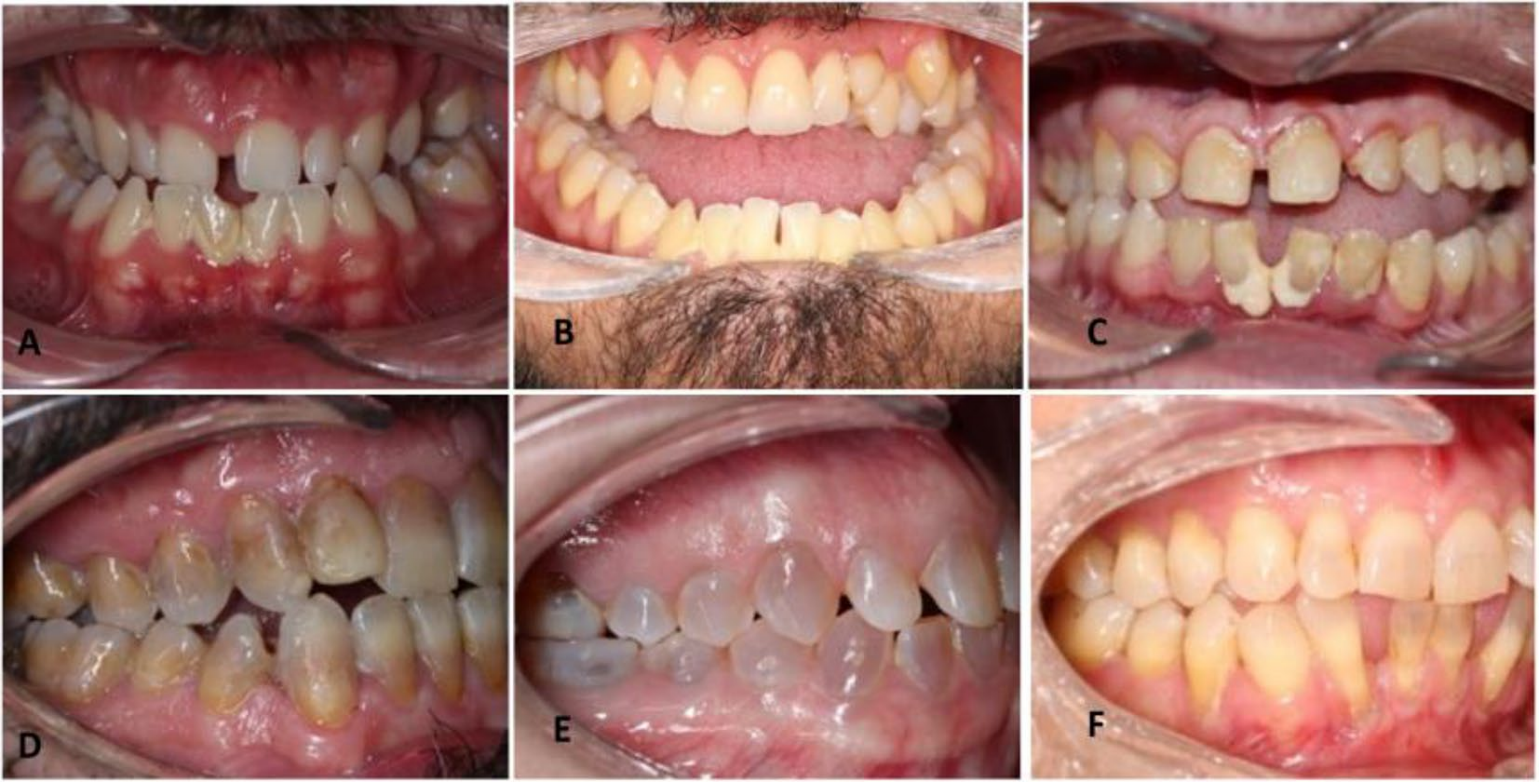
Patients with osteogenesis imperfecta (OI): without dentinogenesis imperfecta (confirmed via periapical radiographs) and with a hypoplastic maxilla that led to anterior and posterior crossbite (type IV) (A); an open bite from the left to right second molar (type III) (B); occlusion only on the right side with a crossbite on the occluding side and poor oral hygiene (type III) (C); OI, dentinogenesis imperfecta and posterior crossbite and tendency to anterior open bite (type IV) (D); normal occlusion with a striking tooth color (type IV) (E); and flaring anteroinferior teeth with marked recission (type IV) (F)

As the research was conducted during the COVID-19 pandemic, clinical participation was very poor (*n* = 25). One patient participated only in the oral examination and was excluded, and the remaining participants (*n* = 24) had the following distributions of type I, III, and IV OI: *n* = 9, 5, and 10, respectively. Only two participants had removable dentures (one with OI type IV and the other with OI type III). One participant with type I OI had one implant. The prevalence of DI was greater in type IV OI (*n* = 5) than in type I and III OI (*n* = 2 and 1, respectively), without any negative influence on OHRQoL (Table 2). There were no significant differences in decayed teeth (DT), ADFT, and OHI between the OI types. However, the number of filled teeth was significantly greater in the type III OI group than in the type I OI group (*p* = 0.029) (Table 3). No significant differences were found between the different types of OI according to the Angle classification or impact on OHRQoL; however, a greater frequency of Class l was found in type I OI, and a greater frequency of Class III, open bite and crossbite was found in type IV and III OI. Figure 2 shows some cases of type IV and type III OI.

**Table 2:** Oral health impact profile questionnaire for different types of OI

OHRQoL	Number of items	Possible range	Observed range	Type I *n* = 20 mean (SD)	Type IV *n* = 30 mean (SD)	Type III *n* = 14 mean (SD)	Total *n* = 64 mean (SD)
OHIP-total	14	0–56	4–51	13.8 [6]	21.4 [12] a	23 [10] b	19.4 (10.8)
OR (CI)	-	-	-	1	1.7(0.3–10)	31 (2-445)*	-
Functional limitation	2	0–8	0–6	0.6 (0.8)	1,5 (1.6)	1,9 (1.5) c	1,3 (1.4)
OR (CI)	-	-	-	1	2 (0.4–10)	15 (1.3–185)*	-
Physical pain	2	0–8	0–8	2.7 (1.5)	3.4 [2]	3.9 (1.7) d	3.3 (1.9)
OR (CI)	-	-	-	1	1,2(0.2-7)	∞*	-
Psychological discomfort	2	0–8	0–8	4.4 (1.5)	5 [2]	5.5 (1.9) e	5 (1.9)
OR (CI)	-	-	-	1	3(0.5–22)	16 (1.6–170)*	-
Physical disability	2	0–8	0–8	1 [1]	2.7 [2, 3]f	3 [1, 9]g	2,4 [2]
OR (CI)	-	-	-	1	5(0.5–53)	59(2.8–1246)*	-
Psychological disability	2	0–8	0–8	2,5 [1, 4]	3.4 [2, 4]	4 (2.4) h	3.2 (2.1)
OR (CI)	-	-	-	1	2(0.4–10)	21(1.7–258)*	-
Social disability	2	0–8	0–8	1 [1]	2.7 [2]i	2 (1.9) j	2 (1.9)
	-	-	-	1	0.2(0.18-2)	*	-
OR (CI)	7,80	11,40	16,33*	0,05			
Handicap	2	0–8	0–8	1 (1.4)	2,4 (1.9) k	2 [2]	2 (1.9)
OR (CI)	-	-	-	1	0.5(0.1-2)	2(0.3–12)*	-

**Statistical analysis**: The Kruskal‒Wallis test was used. The results were confirmed by the Mann‒Whitney U test (nonparametric test). The results are shown as the n or means (SDs). OR (CI) = odds ratio (95% confidence interval). *Statistically significant findings at *p* < 0.05

a) *p* = 0,026 for OI type IV compared to OI type I

b) *p* = 0,007 for OI type III compared to OI type I

c) *p* = 0,010 for OI type III compared to OI type I

d) *p* = 0,042 for OI type III compared to OI type I

e) *P* = 0,016 for OI type III compared to OI type I

f) *p* = 0,034 for OI type IV compared to OI type I

g) *p* = 0,002 for OI type III compared to OI type I

h) *P* = 0,035 for OI type III compared to OI type I

i) *P* = 0,003 for OI type IV compared to OI type I

j) *P* = 0,043 for OI type III compared to OI type I

k) *P* = 0,018 for OI type IV compared to OI type I

**Table 3:** Clinical characteristics of the study population corresponding to DFT and OHI

Independ				
N	5	10	9	24
Decayed teeth	12,10	12,45	12,78	0,97
Filled teeth	7,80	11,40	16,33*	0,05
Adj. DFT (Score)	10,70	11,20	14,94	0,38
Oral hygiene index	14,90	13,80	9,33	0,313
Debris index-S	16,12	13,40	9,11	0,158
Calculus index-S	14,66	14,21	9,72	0,321
OHHDCS^α^	32.5	28.5	38.5	0,235

*Statistical analysis* The Kruskal‒Wallis test was used. The results were confirmed by the Mann‒Whitney U test (nonparametric test). The results are shown as the average range. (αOHHDCS): Oral hygiene habits and dental care survey. * *P* = 0.029 for OI type III compared to OI type I

The total OHIP-14sp score was significantly greater (worse) for OI types IV and III than for type I (p value < 0.05 for both). Type IV OI was associated with higher grades (physical disability *p* = 0.034, social disability *p* = 0.003, and handicap *p* = 0.018) than type I OI. Type III had a greater negative impact on OHRQoL than type I OI and was associated with higher grades of functional limitation (*p* = 0.010), physical pain (*p* = 0.042), psychological discomfort (*p* = 0.016), physical disability (*p* = 0.002), psychological disability (*p* = 0.035), and social disability (*p* = 0.043) (Table 2).

Table 2 shows the results of multivariable-adjusted ordinal logistic regression for adults with OI. A diagnosis of more severe types of OI (types IV and III) was associated with a negative impact on OHRQoL. This association was statistically significant. Compared with type I OI, type IV OI was associated (*p* > 0.05) with a higher grade of physical disability, social disability, and handicap. Type III OI was associated (*p* > 0.05) with a higher score in all domains except the handicap domain (Table 2). After adjusting for sociodemographic variables (age and sex), OI type III compared to OI type I was associated with 31 (95% CI: 2–245) times greater odds of having a higher (worse) OHIP-14 score. This association was predominantly attributed to the strong correlation between OI type III and all domains, including the handicap domain (a subscale of the OHIP). Although the total OHIP-14 score for OI type IV was significantly greater than that for OI type I according to univariate analysis, this difference became statistically nonsignificant after adjusting for the other variables in the model.

## Discussion

This cross-sectional study compared OHRQoL between adults with different types of OI using the OHIP-14sp. The type IV and III OI groups had significantly greater OHIP scores (worse) than did the type I OI group (p values = 0.02 and 0.007, respectively). OHRQoL was worse in type III patients as a result of all the domains being affected except for the handicap domain in comparison with type I OI (p value < 0.05). For participants with type IV OI, the negative impact on OHRQoL was related to higher grades of physical disability, social disability, and handicap compared to type I OI (p value < 0.05). A comparison of our study with the literature was limited, as very little is known about OHRQoL in adults with OI.

Interestingly, the method of administration, based on a questionnaire or interview, did not affect the total OHIP score; however, the interview response rate was significantly greater [29]. Similar results were observed in a study evaluating OHRQoL among children and adolescents with different OI severities living in North America employing the Child Perceptions Questionnaire [12]. In the aforementioned study, there were no differences in OHRQoL among children aged 8–10 years. OHRQoL was significantly lower among adolescents with severe OI (type III) than among those with mild OI (type I). These differences were attributed to the association between OI type III or IV and the functional limitations domain, as shown in our study. Another study evaluated OHRQoL among children with OI using the Child Oral Health Impact Profile-Short Form (COHIP-SF) and demonstrated the deterioration of functional well-being and socioemotional well-being. This difference was related to the severity of OI. Age and sex were not indicators of better or worse OHRQoL in children with OI [30]. The similarities between the patterns observed in both studies and our study on functional limitations repeatedly indicate that functional limitations are a major challenge when assessing OHRQoL in individuals with OI. Gjørup et al. (2021) evaluated OHRQoL in adults with X-linked hypophosphatemia (XLH), and OI was assessed by the OHIP 49-item questionnaire. The median scores for XLH in the domains of functional limitations, pain, psychological discomfort, psychological disability, handicap, and total OHIP significantly exceeded the median scores in the OI group. Individuals with OI types III and IV experienced a greater impact on OHRQoL than individuals with OI type I in only two domains, physical disability and handicap, and no impact on the functional limitation domain was found [31]. The deterioration in OHRQoL in adults with OI, as demonstrated by our results, may serve as a reminder that adults have long-term physical, psychological, and social problems or may be more aware of the oral problems and deterioration caused by this disease. Oral diseases commonly cause complications in all age groups, but this is especially important in adults because oral conditions tend to progress slowly with chronic oral diseases [32]. According to the literature, diabetes worsens OHRQoL, which can lead to functional limitations, physical pain, and psychological discomfort [33]. Patients with asthma exhibit periodontitis, reduced salivary flow, and poorer OHRQoL. This difference is related to the severity of asthma. Patients with mild to moderate and severe asthma had higher grades of physical pain, psychological discomfort, physical disability, and psychological disability than did patients in the no asthma group [34].

Regarding sociodemographic factors, in our study, there were no differences in age, sex, or education level between the different types of OI after performing bivariate analysis; however, there were differences in OHRQoL among people with different education levels. Participants with postgraduate degrees had a better OHRQoL than did those with an education level of high school or less, as the latter was associated with higher grades on the total OHIP-14 and handicap domain. Several studies have shown a negative impact of OHRQoL with increasing age, female sex, and lower education level [35–37]. A negative impact of physical pain and psychological discomfort was found among Canadian adults living in rural areas with secondary or lower education levels [37]. According to the literature, orthodontic and orthognathic surgical interventions are limited in individuals with OI due to the poor quality and quantity of bone and because of the use of bisphosphonate treatment [38]. Diseases affecting the oral cavity have been found to substantially reduce quality of life with increased severity of osteonecrosis related to bisphosphonate treatment [39]. In our study population, 57% (*n* = 24) of the patients were receiving bisphosphonate (19 IV injections and five oral intake) or other drugs related to OI. This highlights the importance of research in this area. Therefore, dentists should be aware of this fact and provide adequate care and attention to these patients through good knowledge, frequent monitoring, and examination.

A limitation of the OHIP-14 is that it does not assess factors influencing OHRQoL, and it is not tailored to the population with OI. These factors could be related to various oral conditions [14]. For this reason, in our study, we described the oral status of 24 adults with different types of OI. According to the literature, people with OI have a greater frequency of malocclusion than does the general population, and the severity of malocclusion is directly proportional to the severity of the disease [40, 41]. In our research, no significant differences were found between the different types of OI according to the Angle classification or its impact on OHRQoL, which is most likely due to the small sample size; however, a greater frequency of Class l was found in type I OI, and greater frequencies of Class III, open bite and crossbite were found in type IV and III OI. Open bite was significantly associated with higher grades of functional limitations (p value = 0.002). Najirad et al. (2020) showed a significant correlation between posterior open bites or crossbites in adolescents with OI and worsening of oral symptoms in the functional limitations domain [16]. Several studies have demonstrated that malocclusion affects the oral function and body image of individuals and can cause psychological disorders [42–44]. The severity of malocclusion is directly related to the impact of the malocclusion on the patient’s quality of life related to oral health [43]. Moreover, the greatest impact was observed in the psychological discomfort and psychological disability domains [44].

In the healthy population, the most common oral health problems are tooth decay and periodontal disease. These conditions have physical, social, and psychological consequences, i.e., they affect the quality of life of patients [45]. For this reason, we analyzed the scores of the adjusted DFT index, which represents the prevalence of caries as a proportion of teeth affected by caries, and we found that the scores of the adjusted DFT index were similar in all the OI groups. A similar finding was observed in a cross-sectional multicenter study describing caries prevalence and experience (CPE) among 319 individuals with OI. In the study, researchers correlated DI with the probability of increased caries experience compared to subjects without DI and controlled for other predictors of CPE [28]. The prevalence of DI in our study was greater in the type IV OI group than in the type I and III OI groups, without any negative influence on OHRQoL. In the literature, there was an increasing prevalence of DI with increased OI severity [46–48]. By analyzing the impact of missing teeth as an influencing factor on OHRQoL in adults with OI, regardless of whether the loss was caused by caries, trauma, or agenesis, we found that more than seven missing teeth were associated with higher grades of physical pain (p value = 0.008); however, no differences were found between different types of OI regarding the number of missing teeth. Previous studies have shown that tooth loss has a negative impact on OHRQoL [13]. A positive quality of life is related to the presence of at least 10 teeth in each arch, preferably natural teeth, and a decrease in the number of teeth deteriorates mastication function. Impaired masticatory performance was associated with lower OHRQoL [49].

Oral hygiene and dental care habits should be taken into consideration. In our study, we found differences between individuals with good and poor oral hygiene index; the latter was associated with higher scores (worse) regarding oral hygiene habits and dental care survey (*p* = 0.004), with no significant difference between OI types or OHRQoL. Previous studies demonstrated a significant association between poor oral hygiene and lower OHRQoL [50].

Unfortunately, our study has several limitations. First, this study was conducted during the COVID-19 pandemic, and participation was very low. Second, this is the first investigation to conduct oral examinations of adults with OI in Spain; therefore, we could not use previous examinations or compare our results in the Spanish population. In the future, we hope to expand the sample size and adjust for other important influencing sociodemographic variables, such as economic status, that may influence the OHRQoL of adults with OI. Another limitation is that we did not analyze the influence of temporomandibular joint problems or periodontal disease on the OHRQoL of adult OI patients. We believe that oral health care for people with OI could be improved by providing a better understanding of the natural history of oral problems in this population. Future research needs to be conducted in this field to overcome all the aforementioned limitations. As a suggestion, future studies should focus on developing questionnaires specific to the OI population.

## Conclusions

In conclusion, this study revealed that moderate (type IV) or severe (type III) OI had a more negative impact on OHRQoL than mild (type I) OI. This is due to the association of moderate OI with the physical disability, social disability, and handicap domains. Severe OI was associated with all domains except for the handicap domain.

### Electronic supplementary material

Below is the link to the electronic supplementary material.

Supplementary Material 1

Supplementary Material 2

Supplementary Material 3

Supplementary Material 4

## Data Availability

Data covered by this agreement (including survey responses, intraoral photographs, and periapical radiographs) were collected during the investigation from participants as part of the project. The data are available at the following links: https://shre.ink/rEGE. https://shre.ink/rEGi.
