# Correction: The impact of osteogenesis imperfecta severity on oral health-related quality of life in Spain: a cross-sectional study

**DOI:** 10.1186/s13023-024-03186-x

**Published:** 2024-04-18

**Authors:** Amira Ahmed Elfituri, Manuel Joaquín De Nova, Mohammadamin Najirad

**Affiliations:** 1 Faculty of Dentistry, Complutense University, Pza. Ramon y Cajal, Moncloa-Aravaca, Madrid, Spain; 2 Department of Orthodontics, Faculty of Dentistry, University of Toronto, Toronto, Canada

**Correction** **:** **Orphanet J Rare Dis 19, 108 (2024)**

**https://doi.org/10.1186/s13023-024-03096-y**

Following publication of the original article [1], we have been notified that Table 3 was missing data in the row “Filled teeth”.

It should be as follows:

Filled teeth 7,80 11,40 16,33* 0,05
