# Supplementary material for: The impact of osteogenesis imperfecta severity on oral health-related quality of life in Spain: a cross-sectional study
Source: Orphanet J Rare Dis. 2024 Mar 8;19:108. doi: 10.1186/s13023-024-03096-y (PMC10921673; doi:10.1186/s13023-024-03096-y)
Supplement: Supplementary file 1 — Supplementary Material 1 [file 13023_2024_3096_MOESM1_ESM.pdf]

Oral health impact profile questionnaire:

1. have you had trouble pronouncing any words because of problems with your teeth, mouth, or dentures?
2. have you felt that your sense of taste has worsened because of problems with your teeth, mouth, or dentures?
3. have you had painful aching in your mouth?
4. have you found it uncomfortable to eat any food because of problems with your teeth, mouth, or dentures?
5. have you been self-conscious because of your teeth, mouth, or dentures?
6. have you felt tense because of problems with your teeth, mouth, or dentures?
7. has your diet been unsatisfactory because of problems with your teeth, mouth, or dentures?
8. have you had to interrupt meals because of problems with your teeth, mouth, or dentures?
9. have you found it difficult to relax because of problems with your teeth, mouth, or dentures?
10. have you been a bit embarrassed because of problems with your teeth, mouth, or dentures?
11. have you been a bit irritable with other people because of problems with your teeth, mouth, or dentures?
12. have you had difficulty doing your usual jobs because of problems with your teeth mouth or dentures?
13. have you felt that life in general was less satisfying because of problems with your teeth, mouth, or dentures?
14. have you been totally unable to function because of problems with your teeth, mouth or dentures?

Cuestionario perfil de impacto de la salud oral, versión española:

1. ¿Ha tenido dificultad para pronunciar palabras?
2. ¿El sabor de sus alimentos ha empeorado?
3. ¿Ha sentido dolor en su boca?
4. ¿Ha presentado molestia al comer?
5. ¿Le preocupan los problemas con su boca?
6. ¿Se ha sentido estresado?
7. ¿Ha tenido que cambiar sus alimentos?
8. ¿Ha tenido que interrumpir sus alimentos?
9. ¿Ha encontrado dificultad para descansar?
10. ¿Se ha sentido avergonzado por problemas con su boca?
11. ¿Ha estado irritable debido a problemas con su boca?
12. ¿Ha tenido dificultad para realizar sus actividades diarias?
13. ¿Ha sentido que la vida en general ha sido menos agradable?
14. ¿Ha sido totalmente incapaz de realizar sus actividades diarias?
