# Supplementary material for: The impact of osteogenesis imperfecta severity on oral health-related quality of life in Spain: a cross-sectional study
Source: Orphanet J Rare Dis. 2024 Mar 8;19:108. doi: 10.1186/s13023-024-03096-y (PMC10921673; doi:10.1186/s13023-024-03096-y)
Supplement: Supplementary file 2 — Supplementary Material 2 [file 13023_2024_3096_MOESM2_ESM.pdf]

Oral hygiene habits and dental care survey

1. How many times a day do you brush your teeth?

Never/ Once a day/ Twice or more. (3,2,1)

2. Have you ever been taught how to brush your teeth?

No/Yes. (1,0)

3. How long does it take to brush your teeth?

I don't brush my teeth/ Less than two minutes/ More than two minutes. (3,2,1)

4. Do you floss or use an interdental brush?

Never/ Sometimes/ Always. (2,1,0)

5. Do you use mouthwash?

Never/ Sometimes/ Always. (2,1,0)

6. How often do you change your toothbrush?

I don't use it/ More than six months/ From four to six months/ From one to three months (3,2,1,0)

7. When you brush your teeth do you brush your tongue?

Never/ Sometimes/ Always. (2,1,0)

8. How many times a year do you go to the dentist?

Never/ One to three times a year/ More than three times a year. (2,1,0)

9. Do you go to the dentist for prevention or when you have any pain?

I don't go to the dentist/ For pain/ prevention. (2,1,0)

10. Do you consider your experience traumatic with the dentist?

No/ Yes. (0,1)

Minimum score 2 and maximum score 21. High values indicate worse oral hygiene and dental care habits.

Encuesta sobre hábitos de higiene bucal y cuidado dental

1. ¿Cuántas veces al día se cepilla los dientes?

Nunca. Una vez al día. Dos veces o más.   (3,2,1)

2. ¿Le han enseñado alguna vez cómo se cepillan los dientes?

No. Sí. (1,0)

3. ¿Cuánto tiempo tarda cepillando los dientes?

No me cepillo los dientes. Menos de dos minutos. Mas de dos minutos. (3,2,1)

4. ¿Usa el hilo dental o el cepillo interdental?

Nunca. A veces. Siempre. (2,1,0)

5. ¿Usa enjuague bucal?

Nunca. A veces. Siempre. (2,1,0)

6. ¿Cada cuándo cambie su cepillo de dental?

No lo uso. Mas de seis meses. De cuatro a seis meses. De uno a tres meses (3,2,1,0)

7. ¿Cuándo se cepilla los dientes se cepilla la lengua?

Nunca. A veces. Siempre. (2,1,0)

8. ¿Cuántas veces al año va al dentista?

Nunca. Una a tres veces al año. Mas de tres veces al año. (2,1,0)

9. ¿Usted va al dentista por prevención o cuando presenta alguna dolencia?

No voy al dentista. Por dolencia. Por prevención. (2,1,0)

10. ¿Considera su experiencia con el dentista traumática?

No. Sí.   (0,1)

Puntuación mínima 2 y máxima 21. Valores altos indican peores hábitos de higiene bucal y cuidado dental.
