# Supplementary material for: The impact of osteogenesis imperfecta severity on oral health-related quality of life in Spain: a cross-sectional study
Source: Orphanet J Rare Dis. 2024 Mar 8;19:108. doi: 10.1186/s13023-024-03096-y (PMC10921673; doi:10.1186/s13023-024-03096-y)

The influence of the severity of osteogenesis imperfect on Oral health-related quality of life, in Spain.

This research forms a part of a doctoral thesis that seeks to get precise information regarding the impact of oral status in patients with osteogenesis imperfecta in relation to physical, psychological, and functional development, reflected in the perception of the well-being of the adult population, the obtained data will be used as an indicator in the future to improve the prognosis, and clinical management decisions of these oral diseases in the affected population.

Who can participate?

People with osteogenesis imperfecta from 18 years old.

Residents in Spain.

SOCIODEMOGRAPHIC QUESTIONS

Before you begin, we would like you to answer some general questions about yourself:

1. Sex
   - Male
   - Female

2. Date of birth ____________________

3. Completed studies
   - No studies
   - Primary or equivalent studies
   - General secondary education
   - Vocational education
   - Higher vocational education
   - University studies
   - Master's or PhD
   - Other

4. Marital status
    - Single
    - Married
    - Separated / Divorced
    - As a couple
    - Widowed

CLINICAL DATA

5. Genetic mutation causing your pathology (osteogenesis imperfecta)
    - COL1A1
    - COL1A2
    - IFITM5
    - SERPINF1
    - CRTAP
    - LEPRE1
    - PPIB
    - SERPINH1
    - FKBP10
    - PLODD2
    - I don't know my genetic mutation
    - I have had the genetic study, but still do not have the results, or I didn't have the genetic test.
6. Are you taking any medications currently related to osteogenesis?
    - Yes
    - No
7. In case of yes indicate which
    - Pamidronate
    - Zoledronate
    - Oral bisphosphonate
    - Prolia

- Costing
- Other

8. Do you take vitamin D?
   - Yes
   - No
9. Do you take calcium?
   - Yes
   - No
10. Do you have any other diseases other than osteogenesis imperfecta?
    - Yes
    - No
11. Indicate which

    ____

12. What are your clinical symptoms?
    - Mild (type I)
    - Moderate (type IV)
    - Severe (type III)

Oral health impact profile questionnaire OHIP-14sp

1. have you had trouble pronouncing any words because of problems with your teeth, mouth, or dentures?
   - Never
   - hardly ever
   - occasionally
   - fairly often

- very often

2. have you felt that your sense of taste has worsened because of problems with your teeth, mouth, or dentures?
    - Never
    - hardly ever
    - occasionally
    - fairly often
    - very often
3. have you had painful aching in your mouth?
    - Never
    - hardly ever
    - occasionally
    - fairly often
    - very often
4. have you found it uncomfortable to eat any food because of problems with your teeth, mouth, or dentures?
    - Never
    - hardly ever
    - occasionally
    - fairly often
    - very often
5. have you been self-conscious because of your teeth, mouth, or dentures?
    - Never
    - hardly ever
    - occasionally
    - fairly often

- very often

6. have you felt tense because of problems with your teeth, mouth, or dentures?
    - Never
    - hardly ever
    - occasionally
    - fairly often
    - very often

7. has your diet been unsatisfactory because of problems with your teeth, mouth, or dentures?
    - Never
    - hardly ever
    - occasionally
    - fairly often
    - very often
8. have you had to interrupt meals because of problems with your teeth, mouth, or dentures?
    - Never
    - hardly ever
    - occasionally
    - fairly often
    - very often

9. have you found it difficult to relax because of problems with your teeth, mouth, or dentures?
    - Never
    - hardly ever
    - occasionally
    - fairly often
    - very often
10. have you been a bit embarrassed because of problems with your teeth, mouth, or dentures?
    - Never
    - hardly ever
    - occasionally
    - fairly often
    - very often
11. have you been a bit irritable with other people because of problems with your teeth, mouth, or dentures?
    - Never
    - hardly ever
    - occasionally
    - fairly often
    - very often
12. have you had difficulty doing your usual jobs because of problems with your teeth mouth or dentures?
    - Never
    - hardly ever
    - occasionally
    - fairly often

- very often

13. have you felt that life in general was less satisfying because of problems with your teeth, mouth, or dentures?
    - Never
    - hardly ever
    - occasionally
    - fairly often
    - very often
14. have you been unable to function because of problems with your teeth, mouth, or dentures?
    - Never
    - hardly ever
    - occasionally
    - fairly often
    - very often

Oral hygiene habit and dental care survey:

1. How many times a day do you brush your teeth?

- Never.
- Once a day.
- Twice or more.

2. Have you ever been taught how to brush your teeth?

- No.
- Yes.

3. How long does it take to brush your teeth?

- I don't brush my teeth.
- Less than two minutes.
- More than two minutes.

4. Do you floss or use an interdental brush?

- Never.
- Sometimes.
- Always.

5. Do you use mouthwash?

- Never.
- Sometimes.
- Always.

6. How often do you change your toothbrush?

- I don't use it.
- More than six months.
- From four to six months.
- From one to three months.

7. When you brush your teeth do you brush your tongue?

- Never.
- Sometimes.
- Always.

8. How many times a year do you go to the dentist?

- Never.
- One to three times a year.
- More than three times a year.

9. Do you go to the dentist for prevention or when you have any pain?

- I don't go to the dentist.
- For pain.
- For prevention.

10. Do you consider your experience traumatic with the dentist?

- No.
- Yes.

LRPD

In compliance with current legal regulations on the Protection of personal data, you are informed that your data will be processed to promote as many activities and initiatives contribute to the personal, cultural, and human development of people with osteogenesis imperfecta, achieve their social integration and work and improve the quality of life for them and their families, as well as, where appropriate, the realization of medical tests incorporated into research projects. In this case, if you do not provide your data, we will not be able to fulfill the intended purpose. We inform you that the legal basis for the processing of your data is the consent of the interested party.

The data provided will be kept as long as the commercial relationship is maintained or during the years necessary to comply with the Legal obligations.

Another purpose is to send you informative and courtesy communications related to our entity, through telephone, ordinary postal mail, email, or equivalent means of electronic communication. Check the appropriate box that applies to receive informative communications.

- ○ Yes
- ○ No

Accept and consent to their treatment by THE RESPONSIBLE who informs you that your data will be processed by the professionals collaborating with this center and guarantees that the data collected will not be transferred to third parties or used for any purpose other than the one for which they have been collected. THE RESPONSIBLE has adopted all the necessary technical and organizational measures to guarantee the security and integrity of the personal data it processes, as well as to avoid its loss, alteration, and/or access by unauthorized third parties. You are informed that you can exercise the rights of access, rectification, deletion, and, where appropriate, opposition and portability of the data, as well as the limitation of treatment by sending a request accompanied by an identification document to the indicated address in the heading of this document. Likewise, you are also informed that, if you do not obtain satisfaction in the exercise of your rights, you can exercise your right to file a claim before a Competent Data Protection, currently the Spanish Data Protection Agency.

Intraoral photo and periapical radiograph.

Participant 1: EXCEL response 47. Date of birth: 29/03/1978.

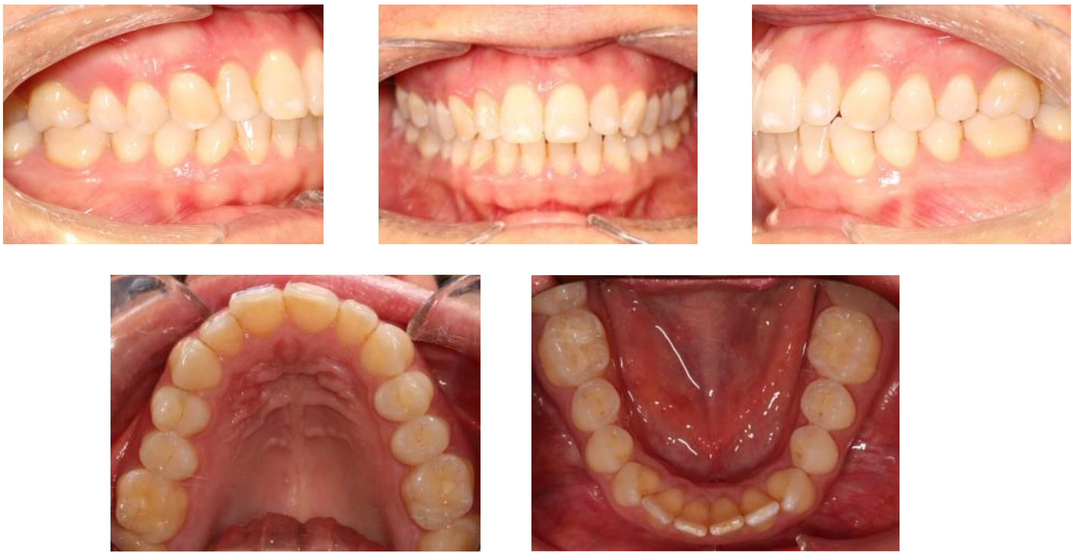

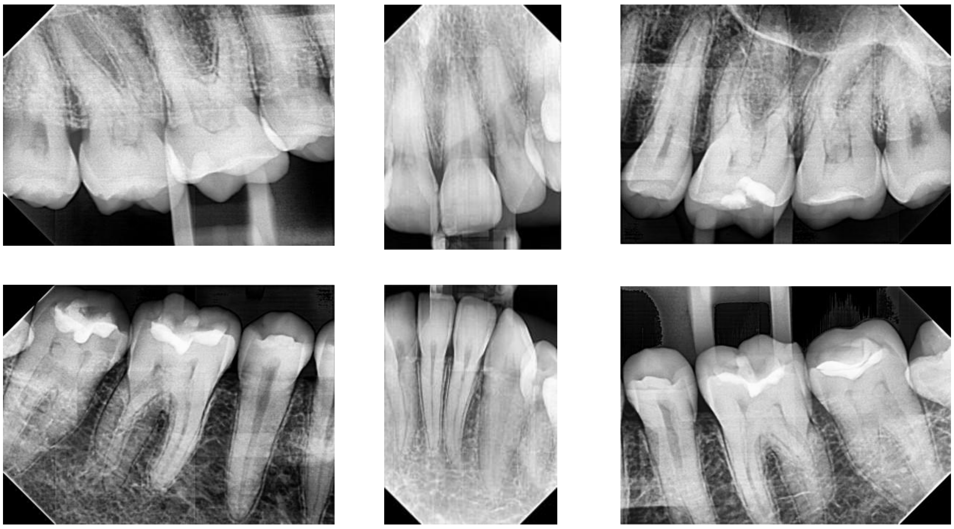

Participant 2: EXCEL response 53 1/05/1988.

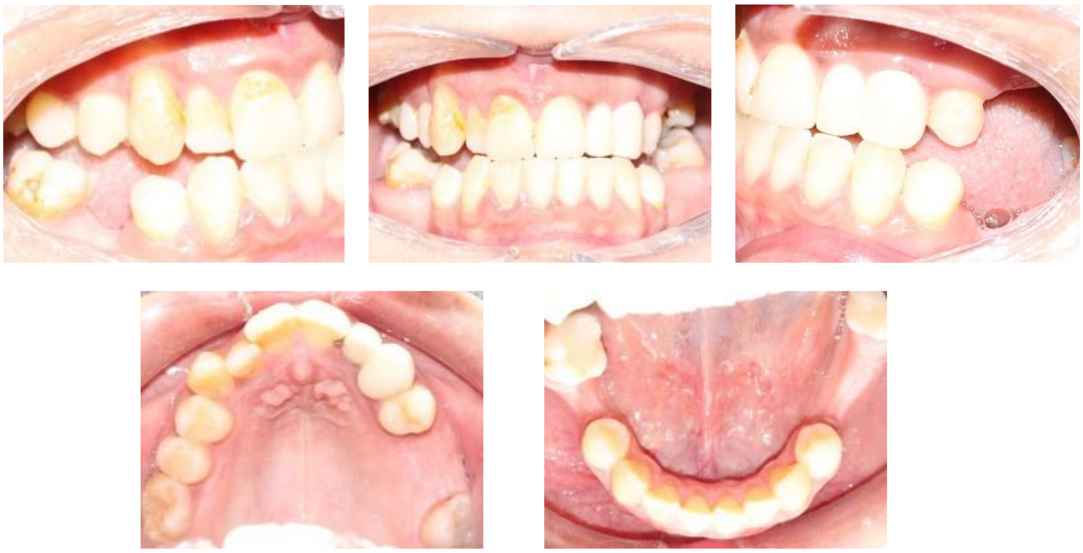

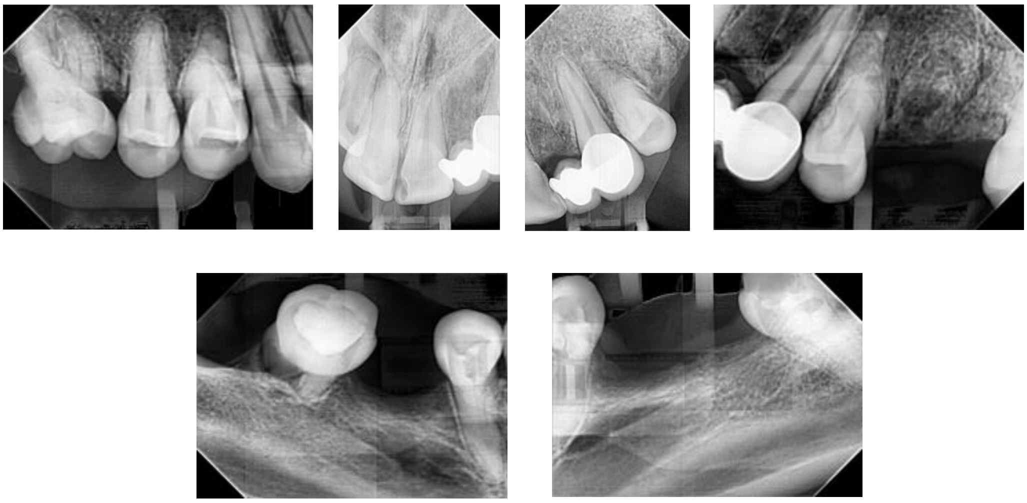

Participant 3: EXCEL response 46 16/07/1974.

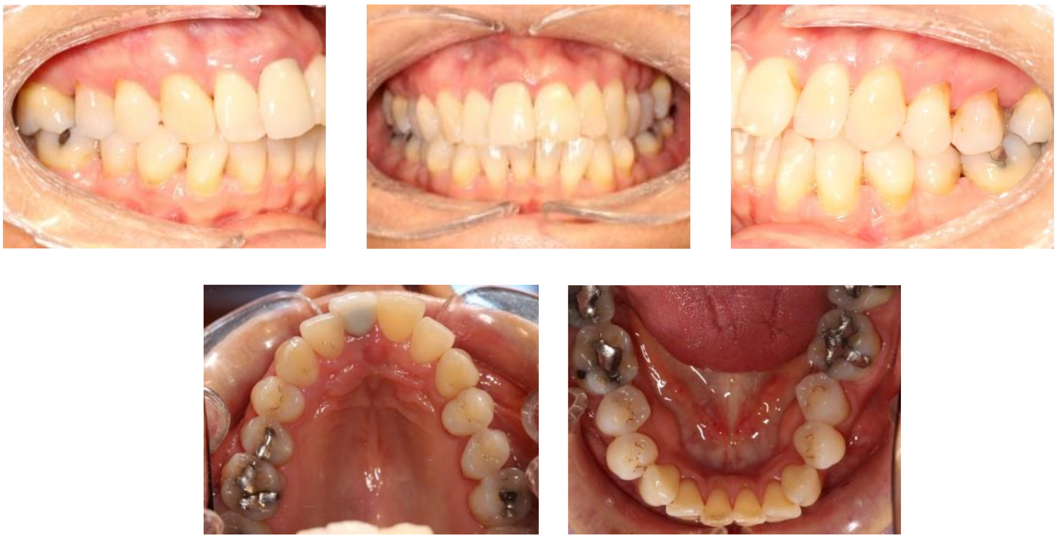

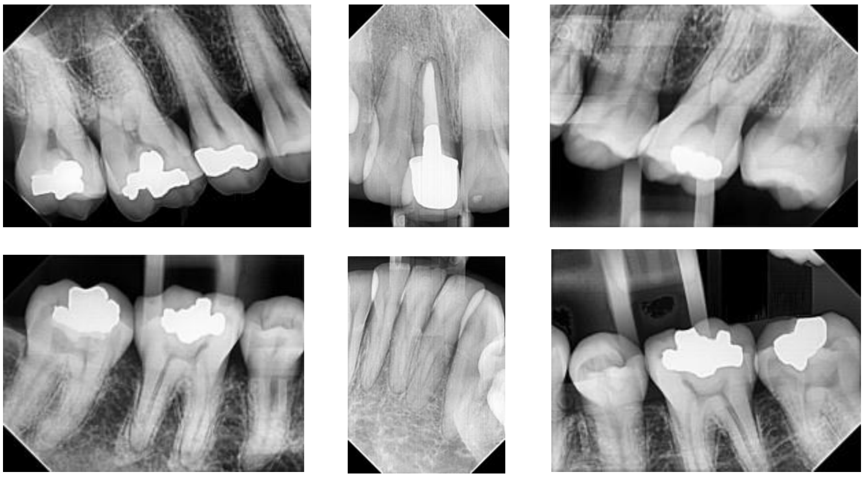

Participant 4: Excel response 6 21/03/1978.

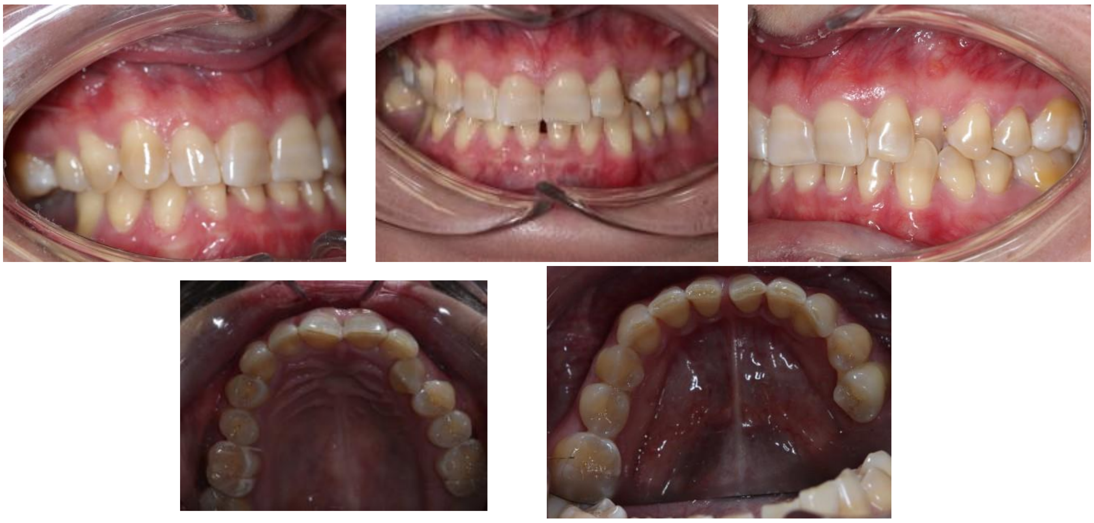

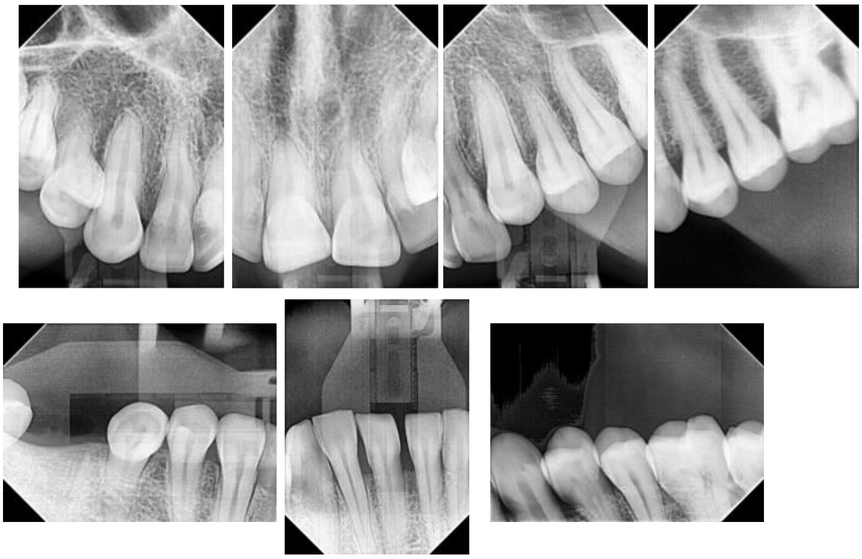

Participant 5: Excel response 49 29/08/1982

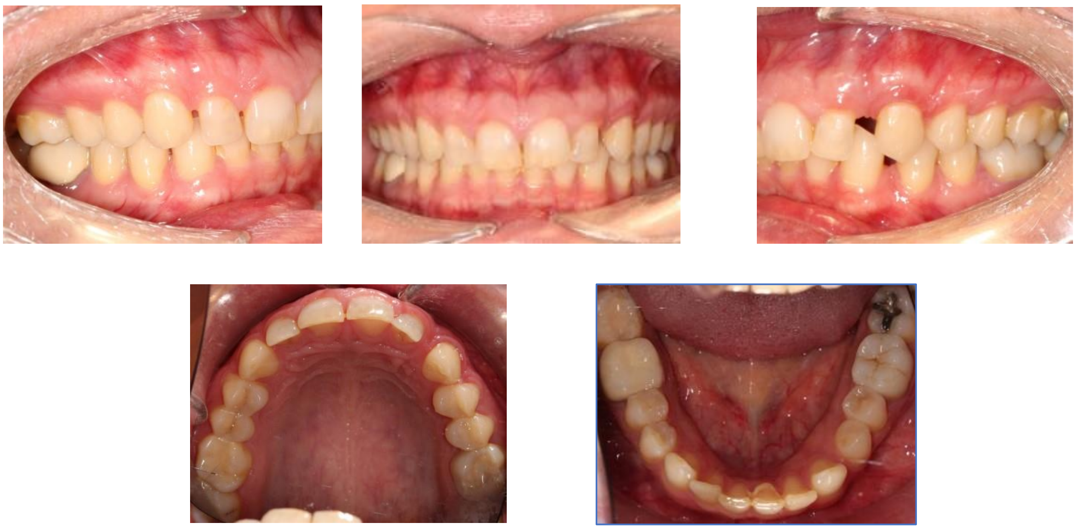

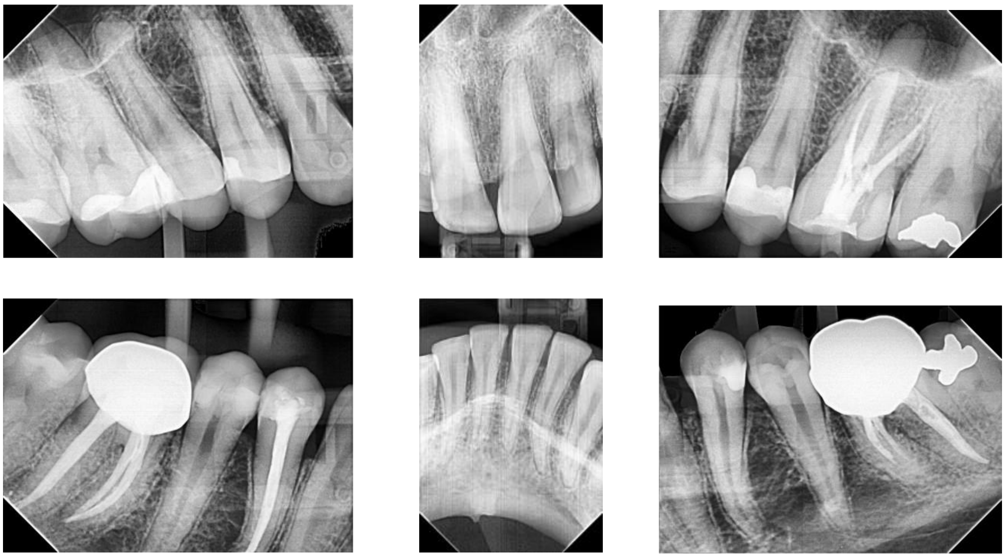

Participant 6: Excel response 17 23/01/1990

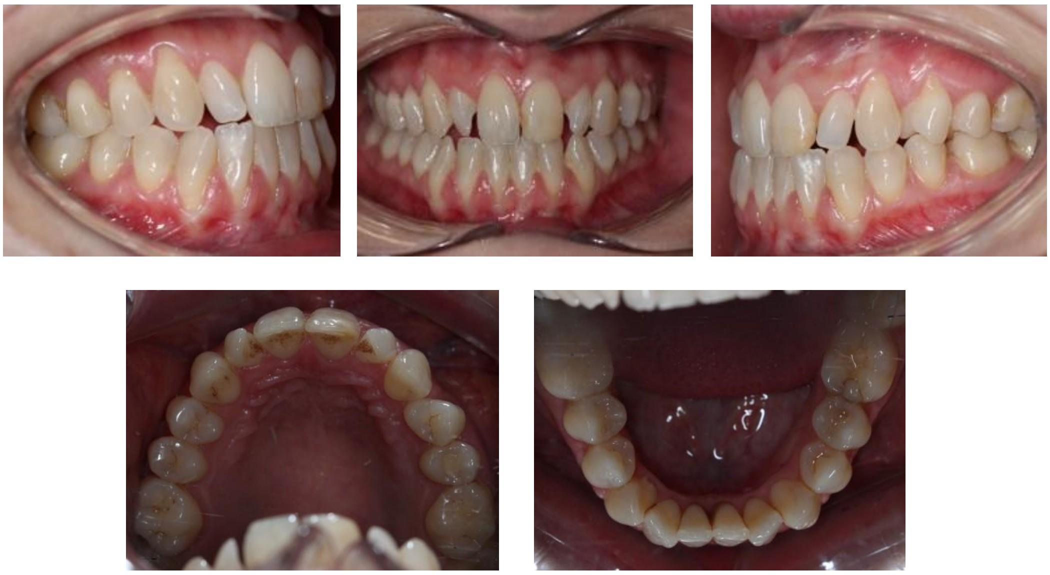

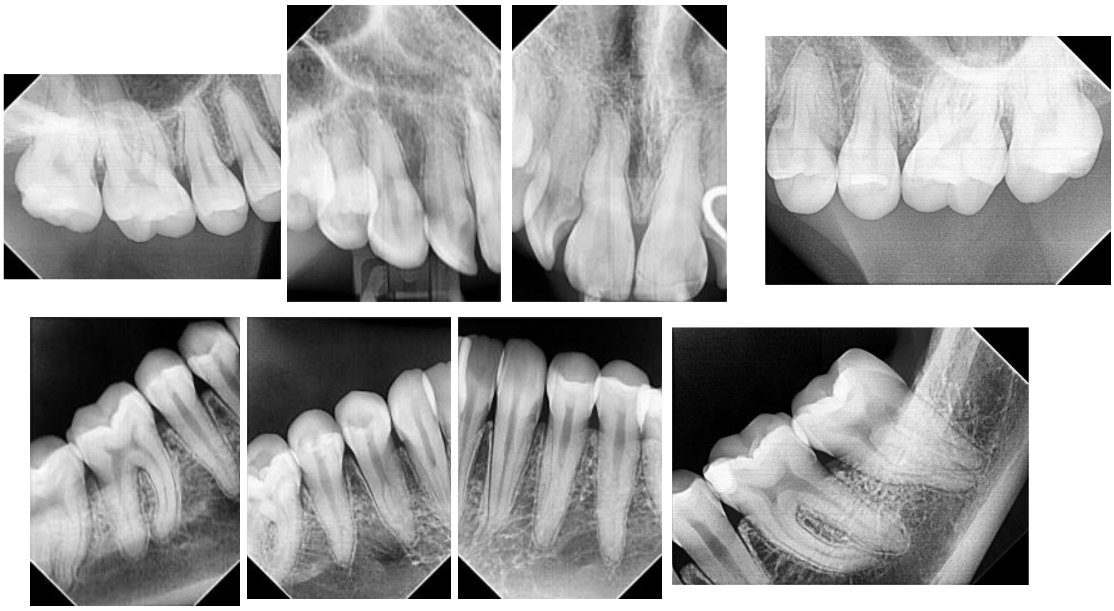

Participant 7: **Excel response 58** **16/03/1970**

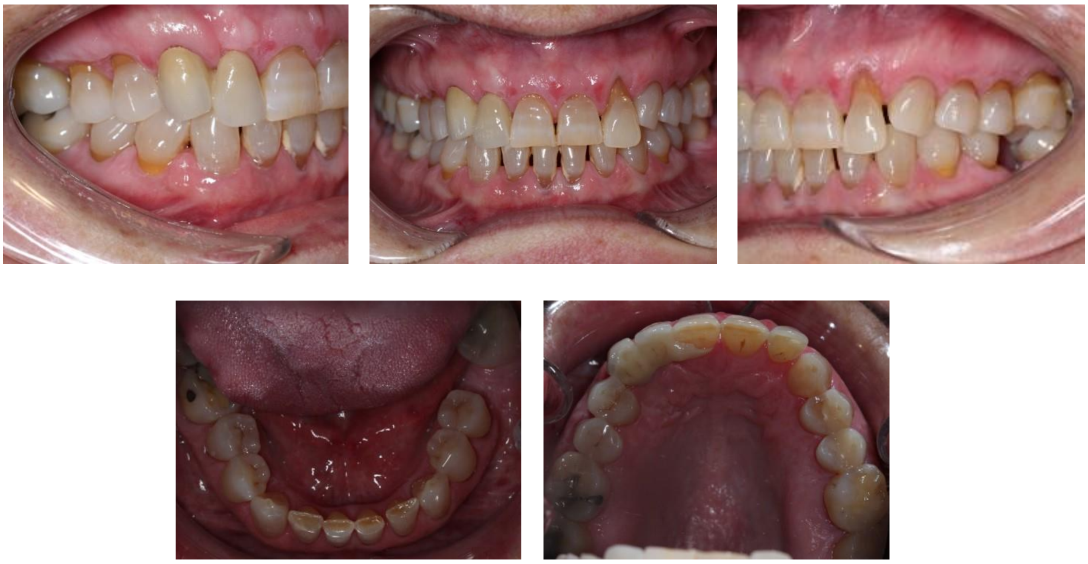

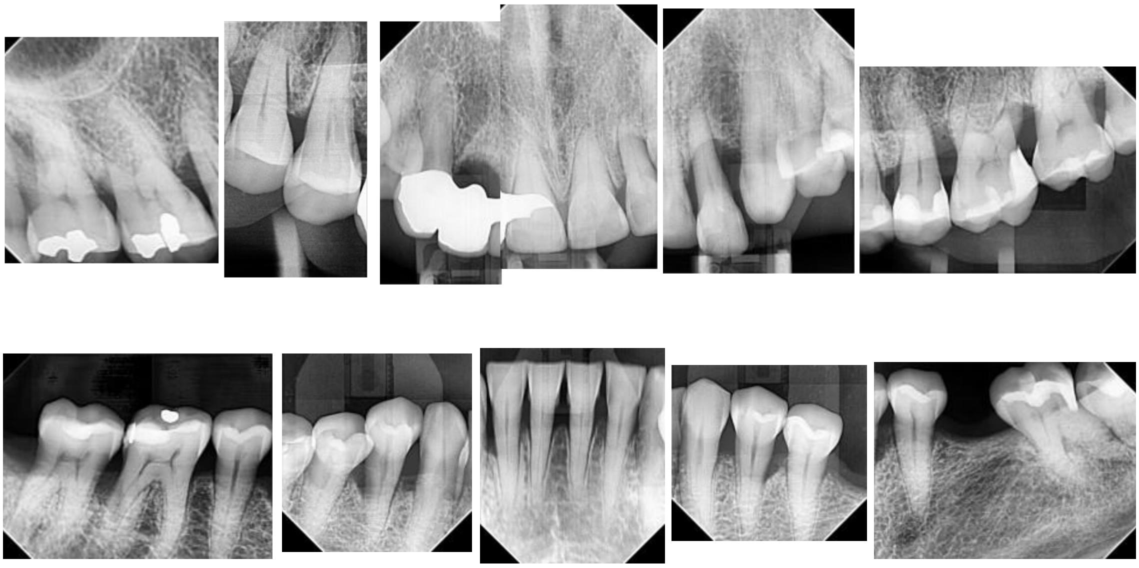

Participant 8: **Excel response 59** **20/02/1979**

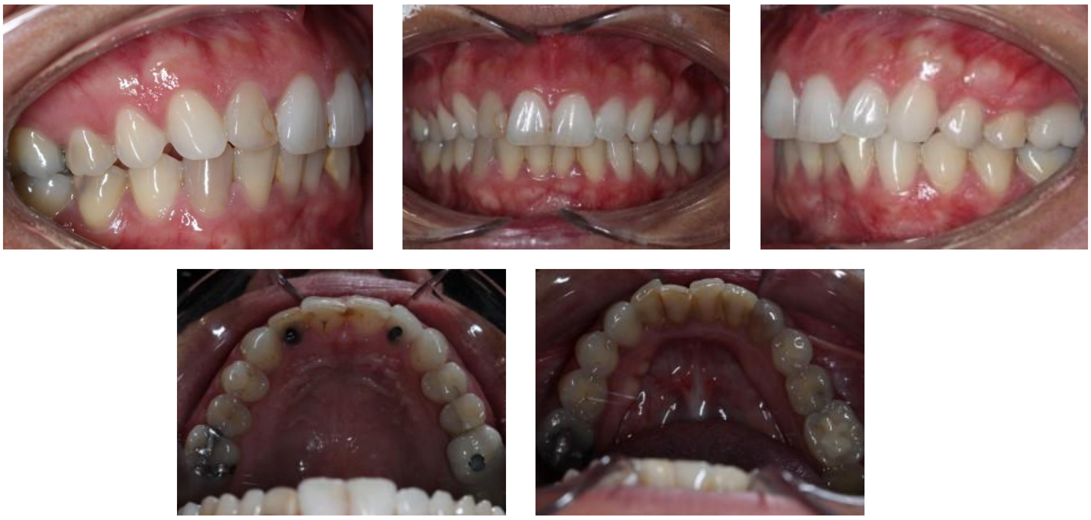

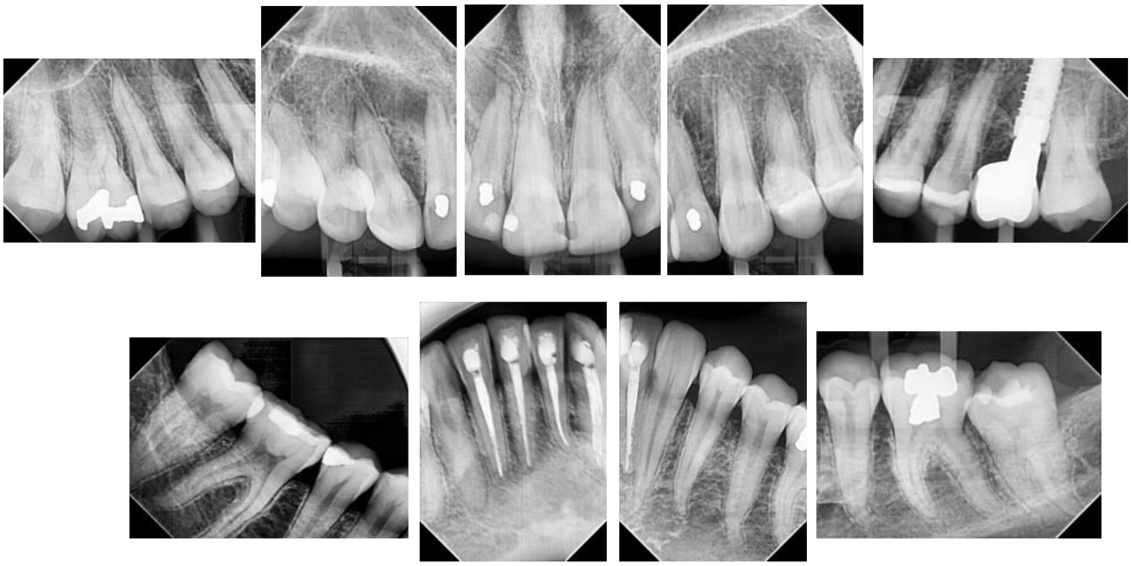

Participant 9: **Excel response 55** **20/05/1972**

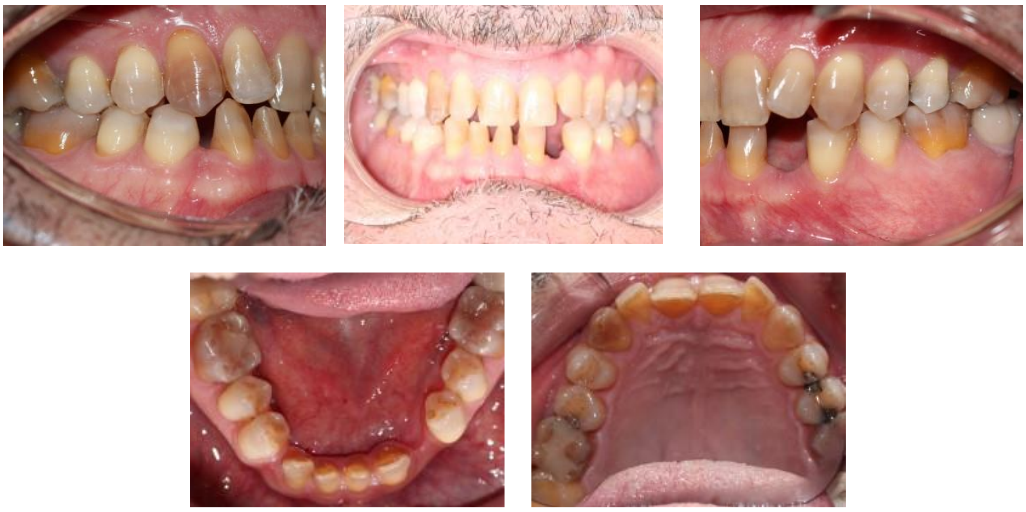

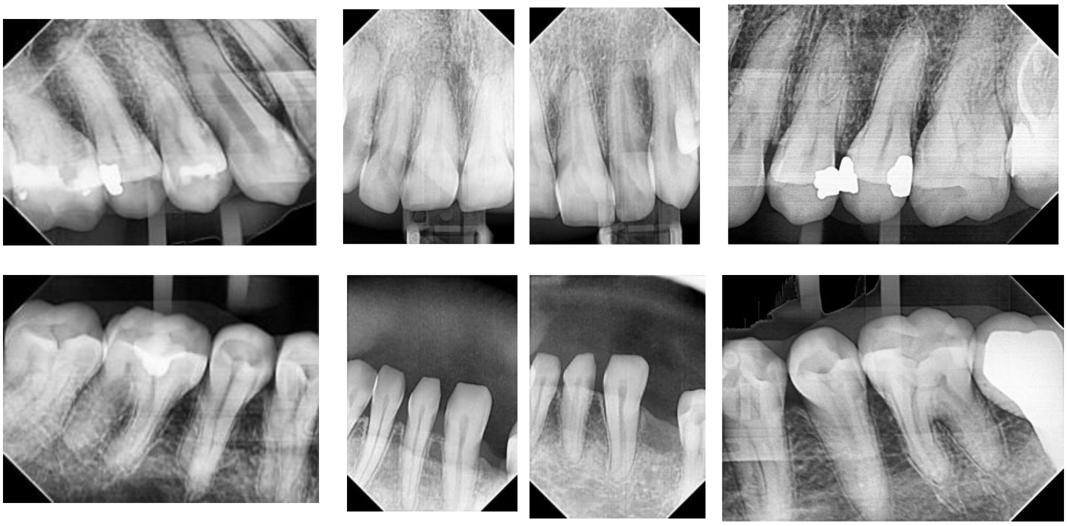

Participant 10: Excel response 57 29/09/1969.

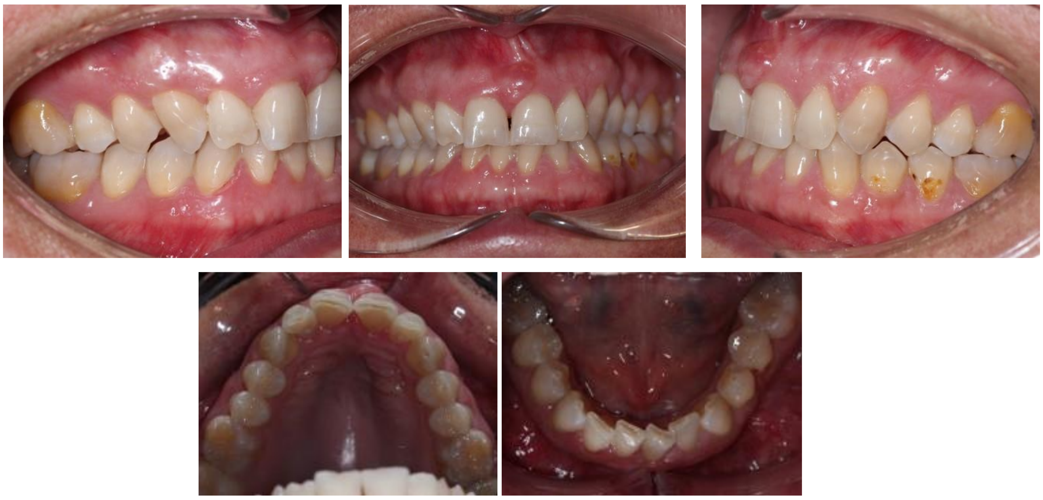

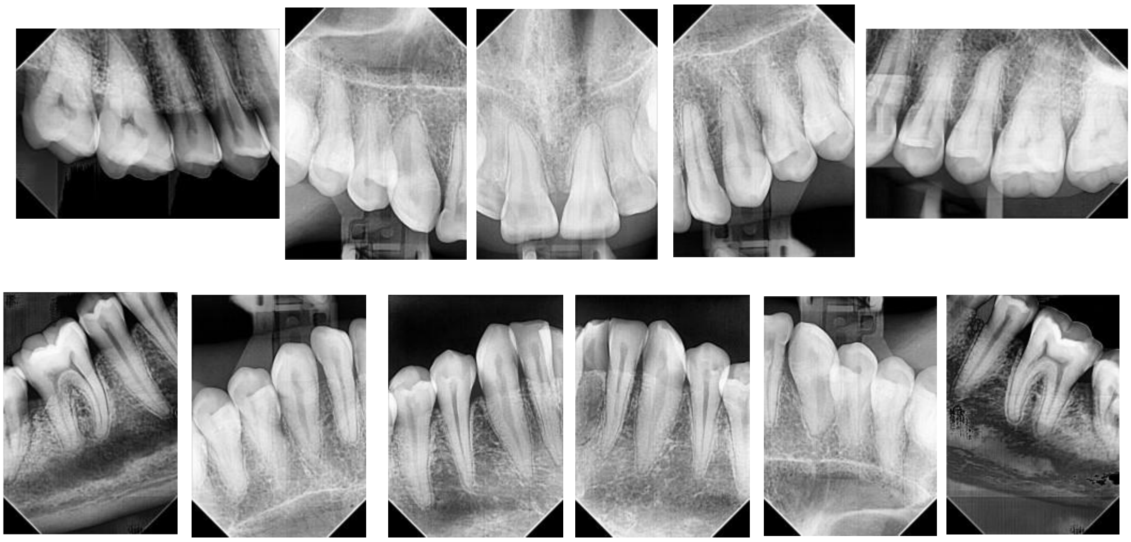

Participant 11: Excel response 54 24/04/1963.

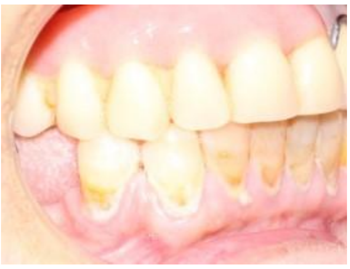
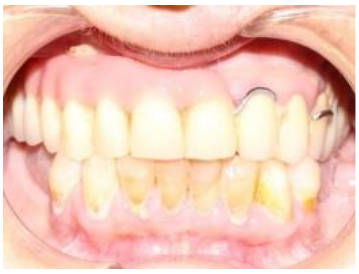
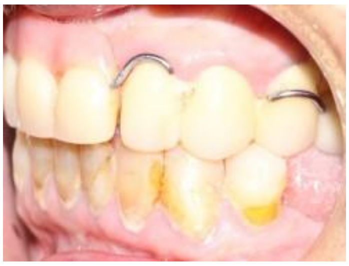
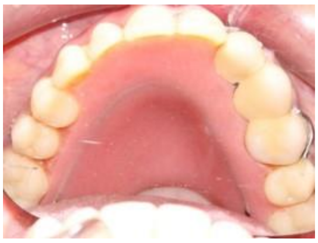
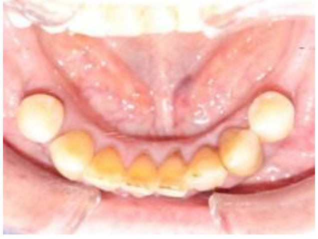
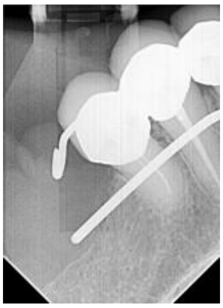
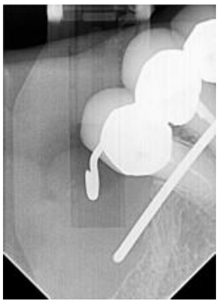
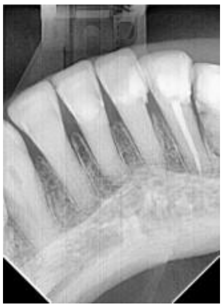
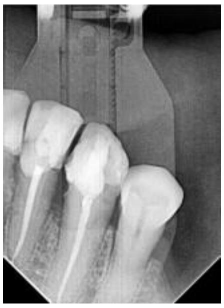

Participant 12: Excel response 64 21/01/1996.

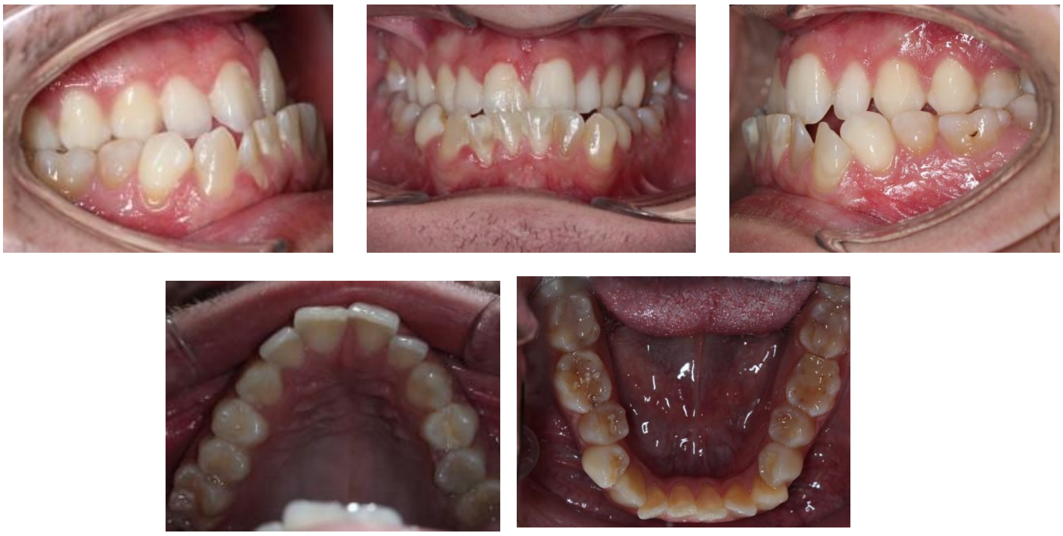

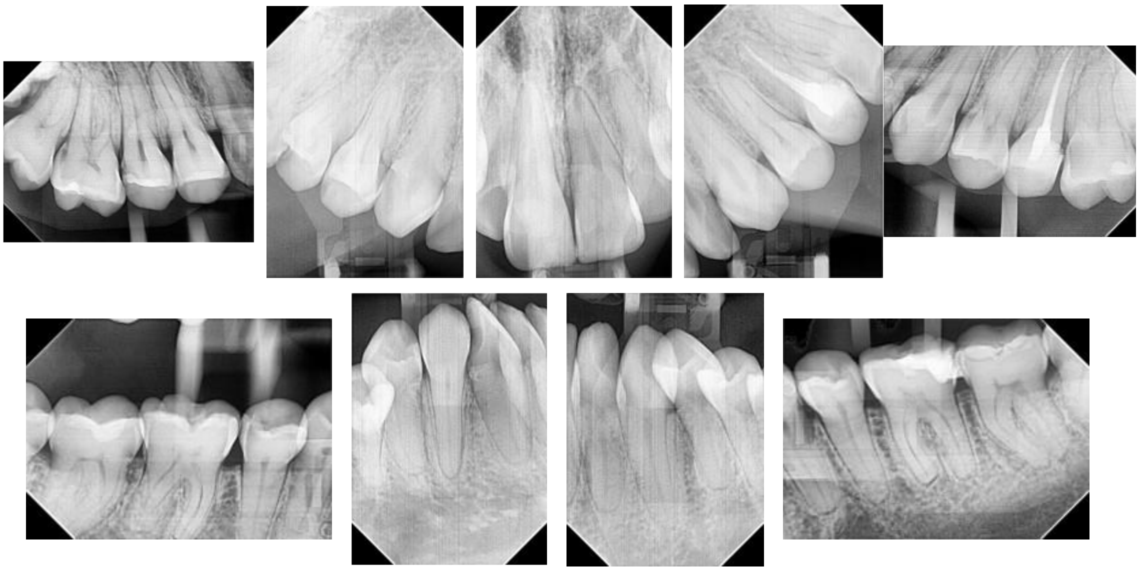

Participant 13: **Excel response 62** **11/05/1996**

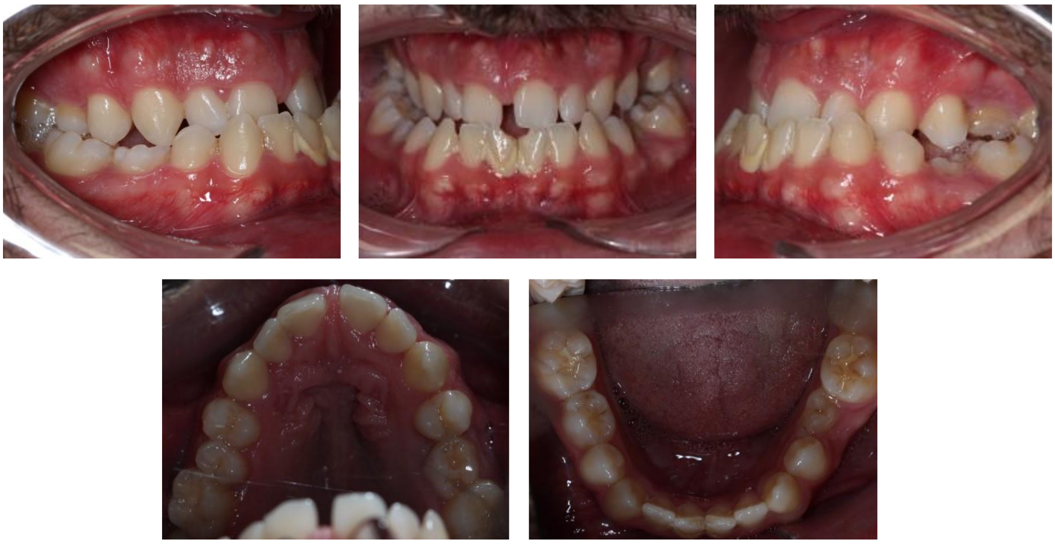

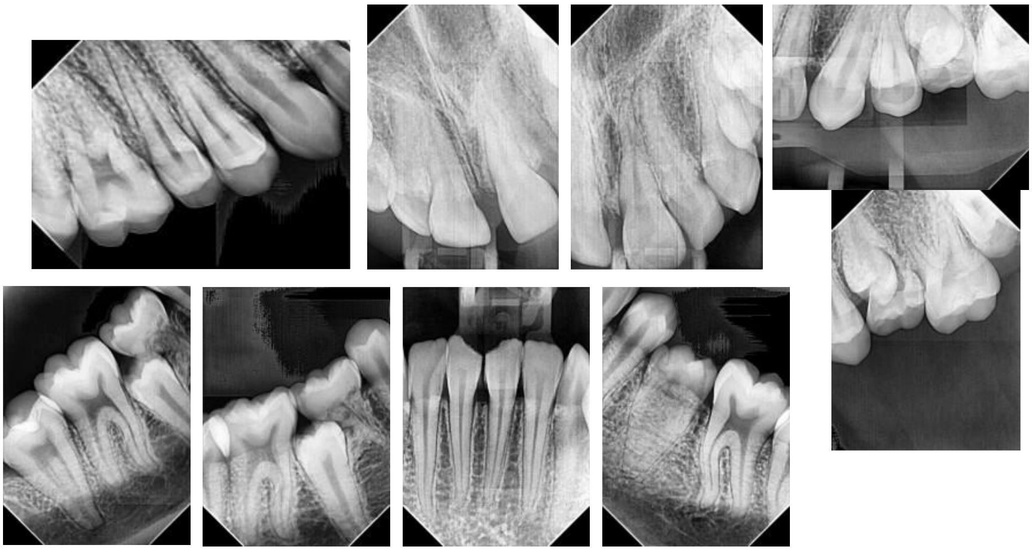

Participant 14: EXCEL response 65 01/05/1968.

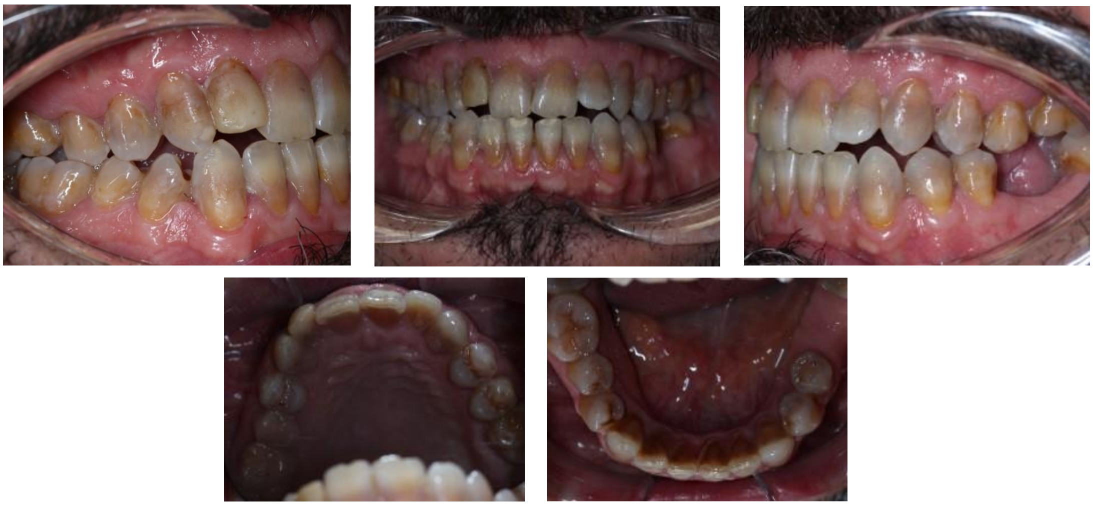

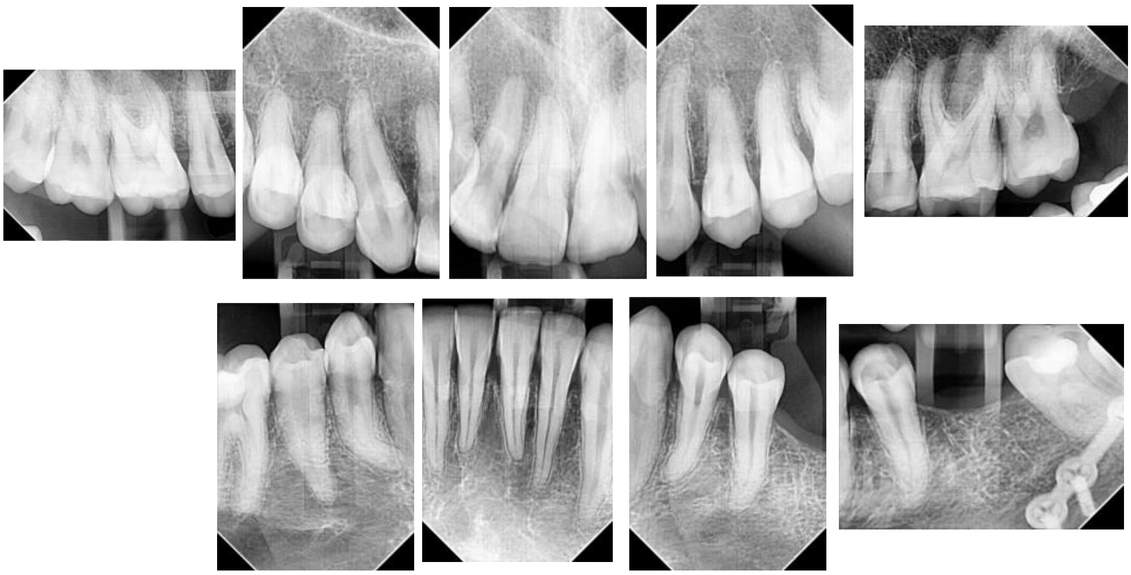

Participant 15:   Excel response 52   11/04/1960.

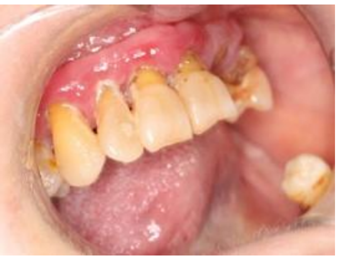
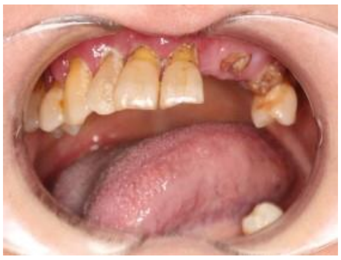
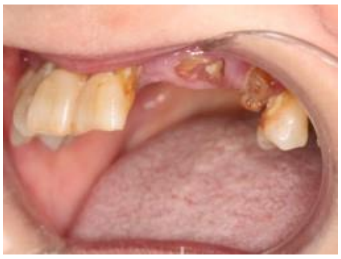

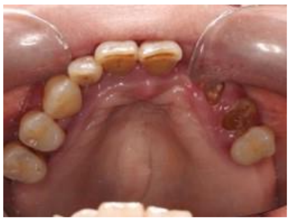
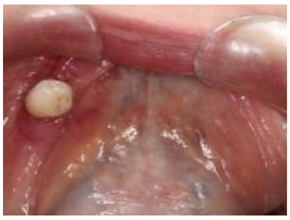

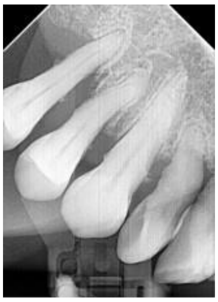
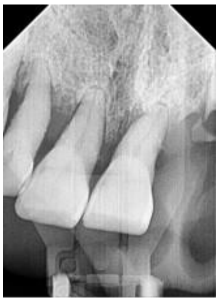
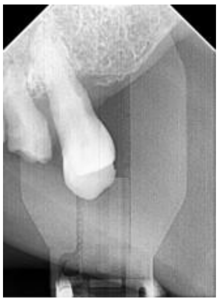

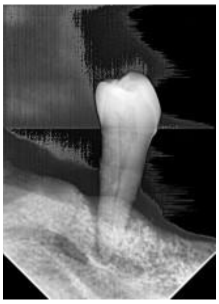

Participant 16: Excel response 61 23/02/2001

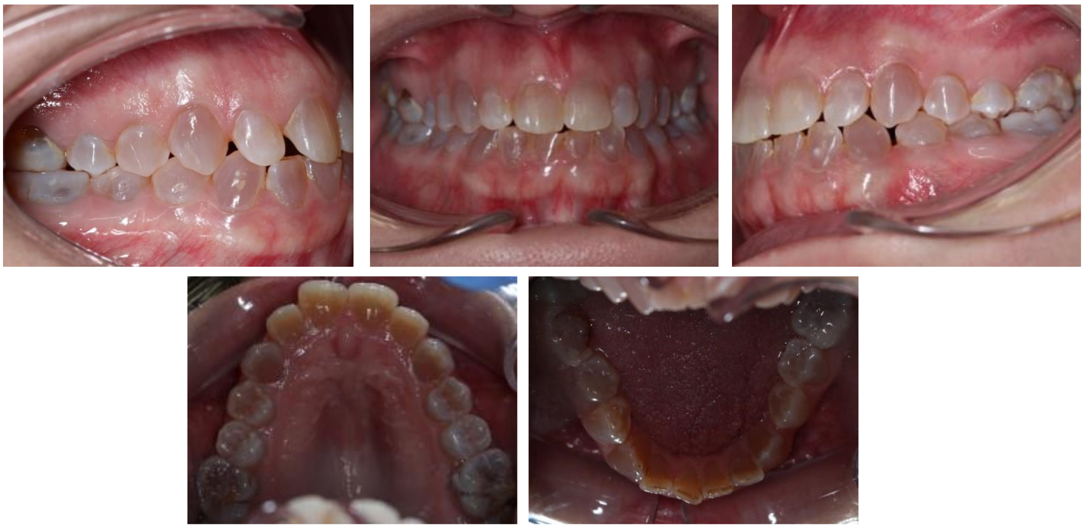

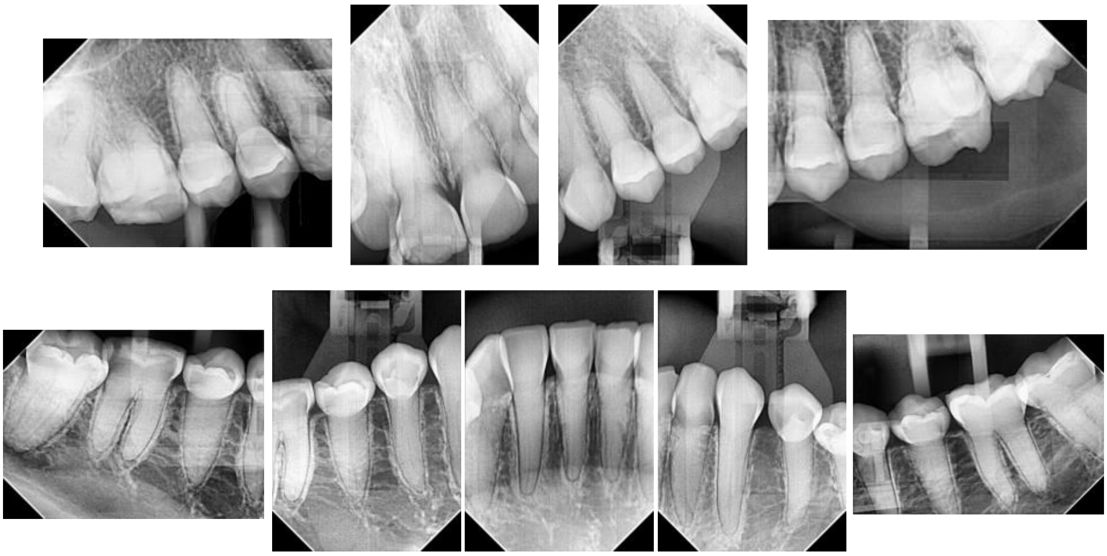

Participant 17: EXCEL response 48 3/05/1958

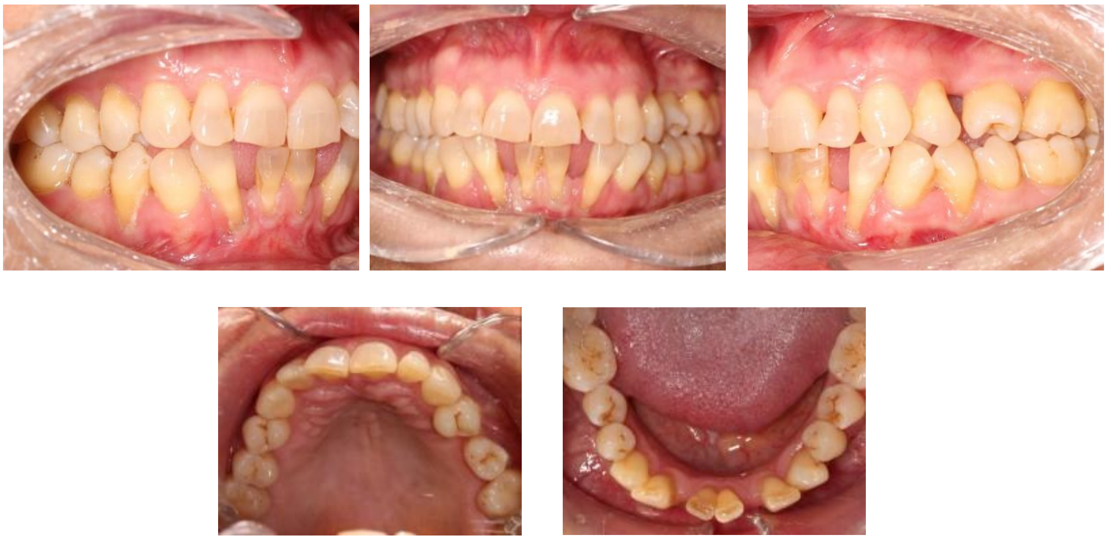

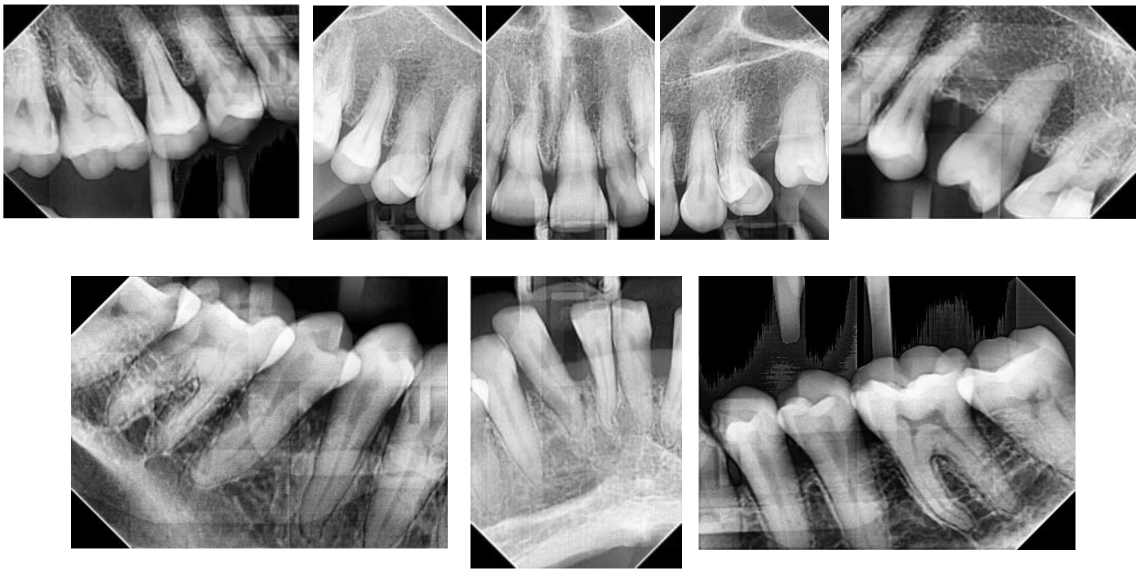

Participant 18: Excel response 60 10/11/1965.

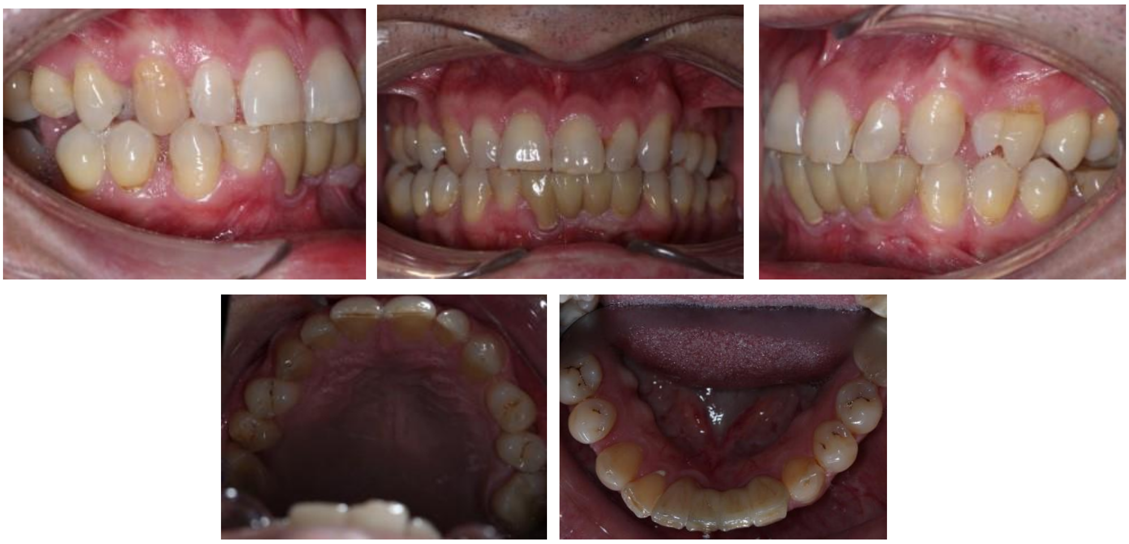

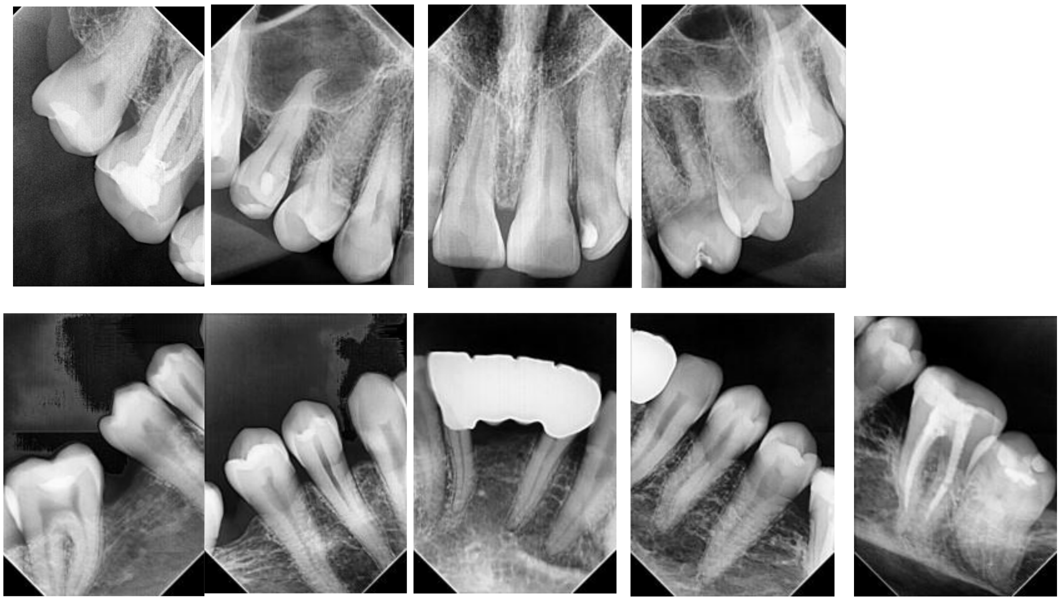

Participant 19:   Excel   45   4/08/1982

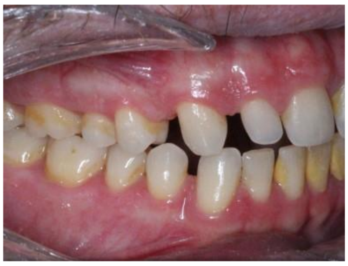
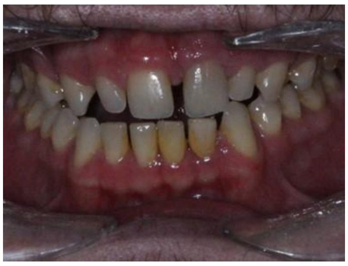
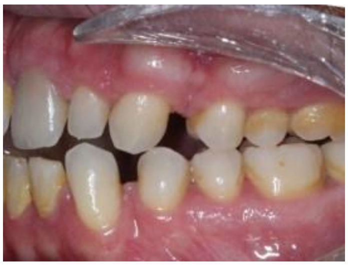

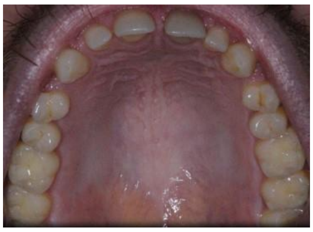
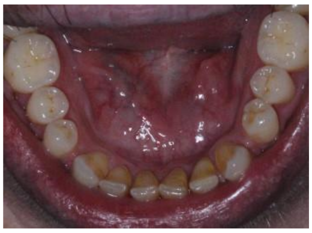

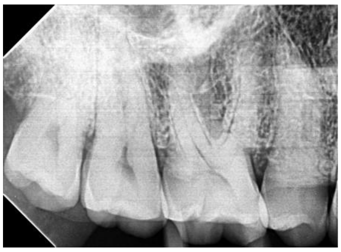
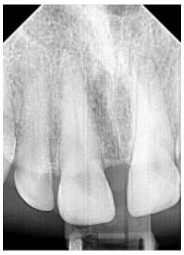
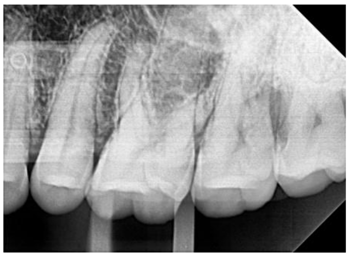

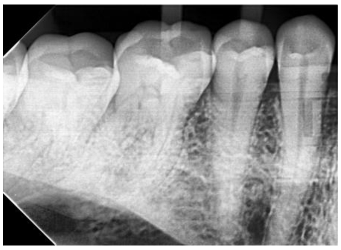
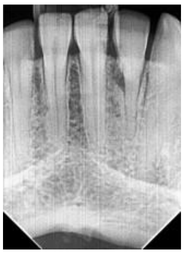
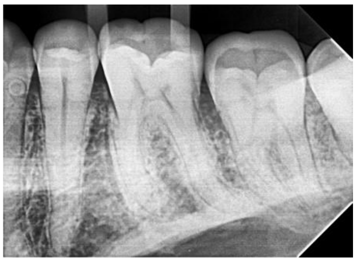

Participant 20: **EXCEL response 50** **1/08/1967.**

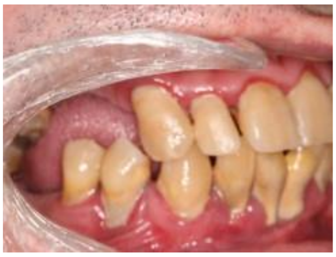
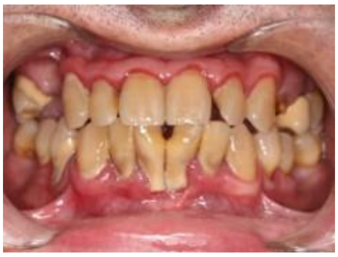
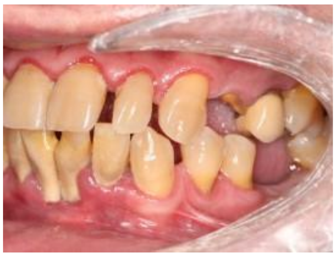
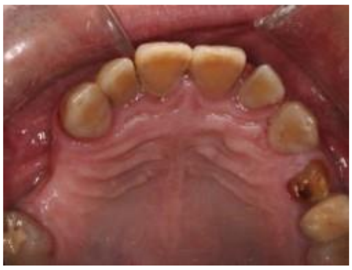
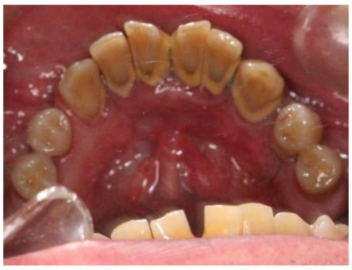

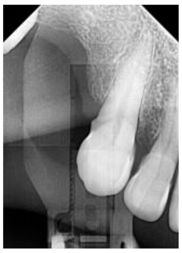
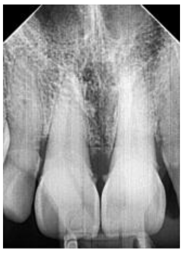
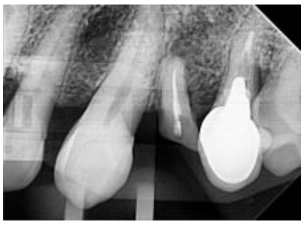
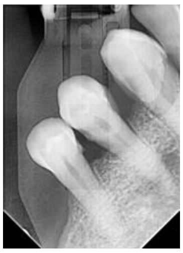
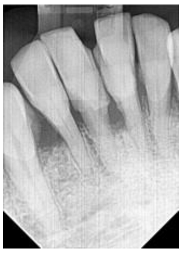

Participant 21: EXCEL response 51 15/08/1963.

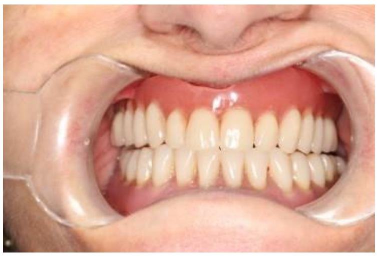

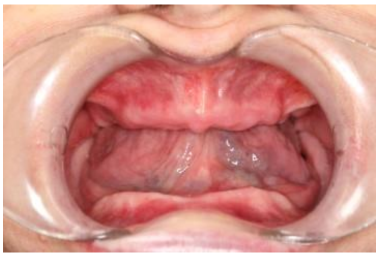

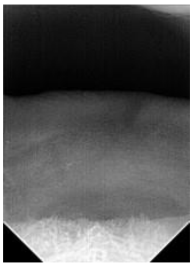

Participant 22: EXCEL response 56 30/11/1976

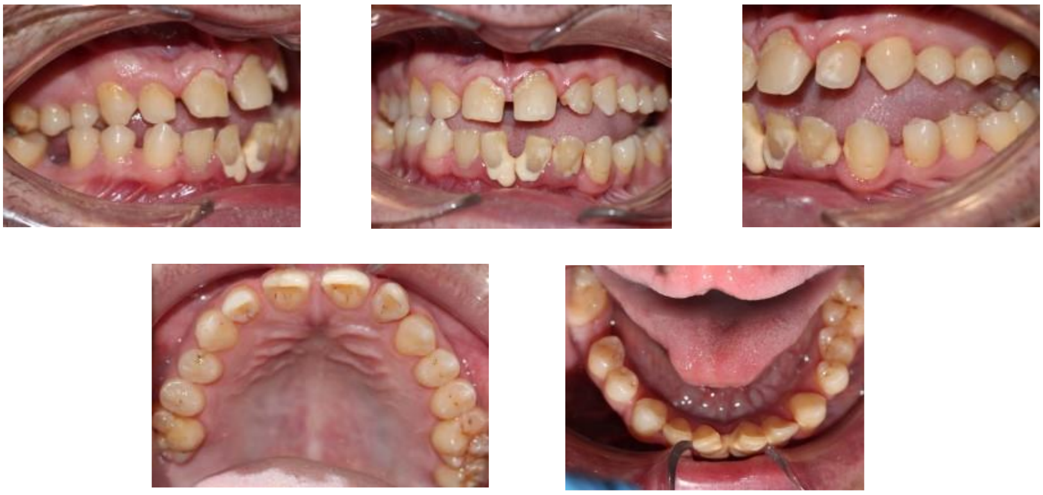

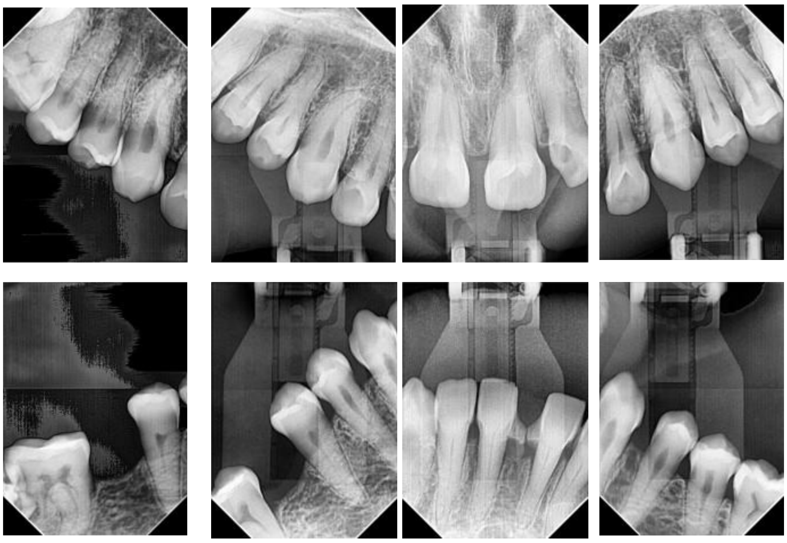

Participant 23: Excel response 2 21/8/85

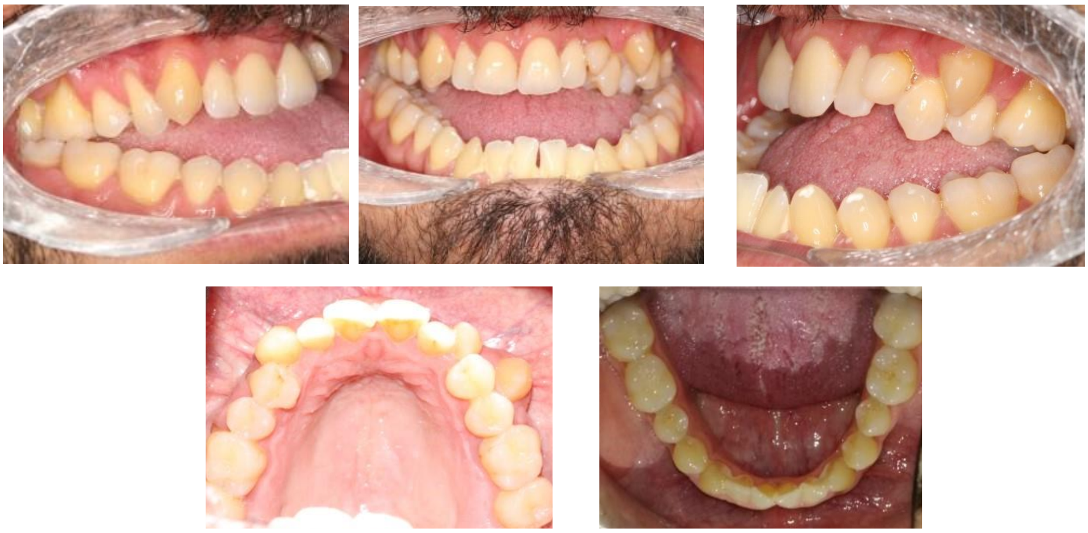

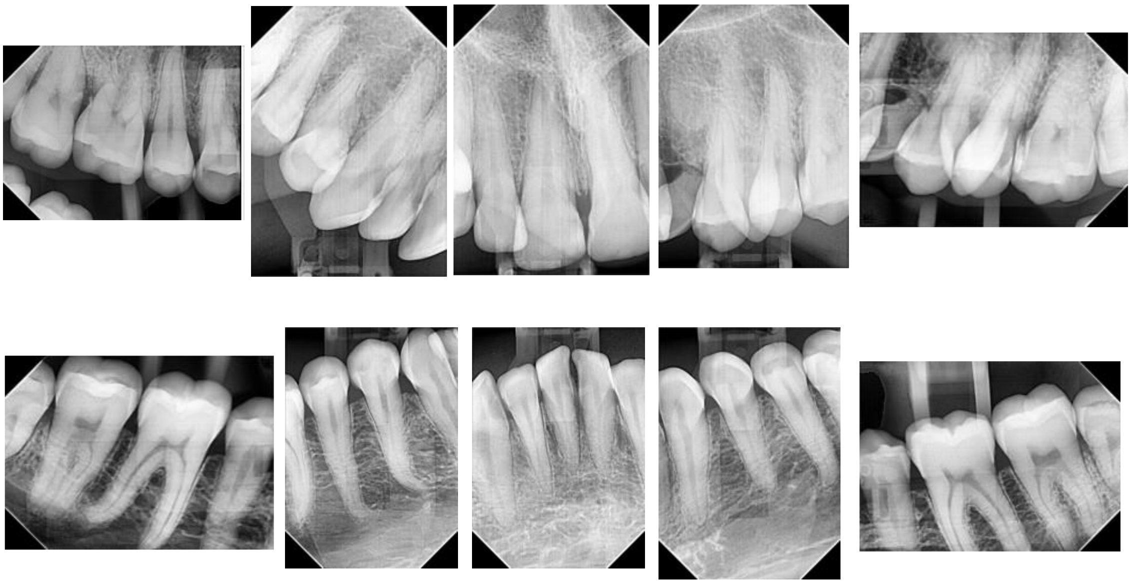

Participant 24: **Excel response 44** **5/01/1977**

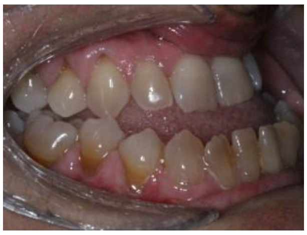
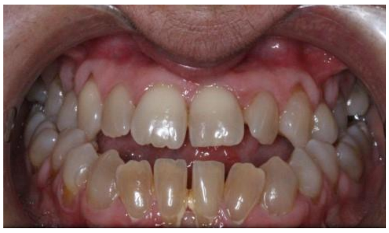
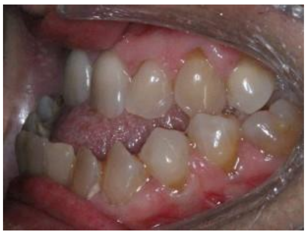
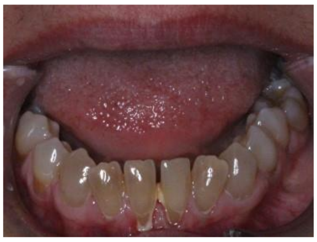
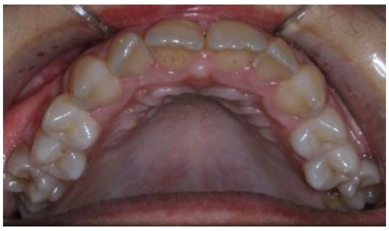

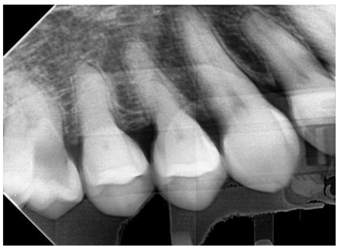
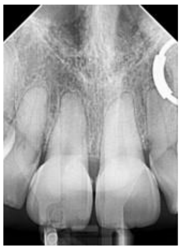
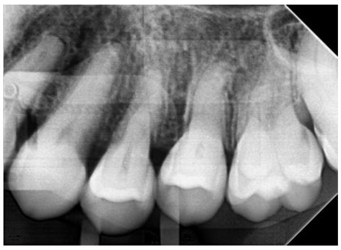
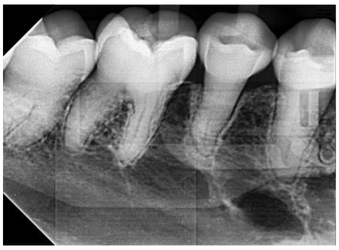
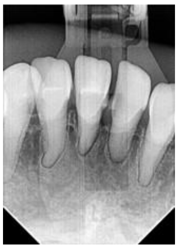
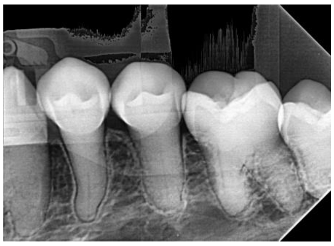

Supplement: Supplementary file 3 — Supplementary Material 3 [file 13023_2024_3096_MOESM3_ESM.pdf]
